# Evolution of Local Structural Motifs in Colloidal Quantum Dot Semiconductor Nanocrystals Leading to Nanofaceting

**DOI:** 10.1021/acs.nanolett.2c04851

**Published:** 2023-03-13

**Authors:** Bo Hou, Felix Cosmin Mocanu, Yuljae Cho, Jongchul Lim, Jiangtao Feng, Jingchao Zhang, John Hong, Sangyeon Pak, Jong Bae Park, Young-Woo Lee, Juwon Lee, Byung-Sung Kim, Stephen M. Morris, Jung Inn Sohn, SeungNam Cha, Jong Min Kim

**Affiliations:** † School of Physics and Astronomy, Cardiff University, Queen’s Building, The Parade, Cardiff, Wales CF24 3AA, United Kingdom; ‡ Laboratoire de Physique de l’École Normale Supérieure, ENS, Université PSL, CNRS, Sorbonne Université, Université de Paris, 75005 Paris, France; § Department of Engineering Science, University of Oxford, Parks Road, Oxford OX1 3PJ, United Kingdom; ∥ University of Michigan−Shanghai Jiao Tong University Joint Institute, Shanghai Jiao Tong University, 800 Dong Chuan Road, Minghang District, Shanghai 200240, China; ⊥ Graduate school of energy science and technology, Chungnam National University, Daejeon 34134, Republic of Korea; # Department of Environmental Science & Engineering, School of Energy and Power Engineering, Xi’an Jiaotong University, Xi’an 710049, China; g Microsoft Corporation, Redmond, Washington 98073, United States; h School of Materials Science and Engineering, Kookmin University, Seoul 02707, Republic of Korea; i School of Electronic and Electrical Engineering, Hongik University, Seoul 04066, Republic of Korea; j Division of Physics and Semiconductor Science, Dongguk University-Seoul, Seoul 04620, Republic of Korea; k Department of Physics, Sungkyunkwan University, Suwon, Gyeonggi-do 16419, Republic of Korea; l Department of Engineering, Electrical Engineering Division, 9 JJ Thomson Avenue, University of Cambridge, Cambridge CB3 0FA, United Kingdom

**Keywords:** quantum dots, morphology, electron microscopy, density functional theory

## Abstract

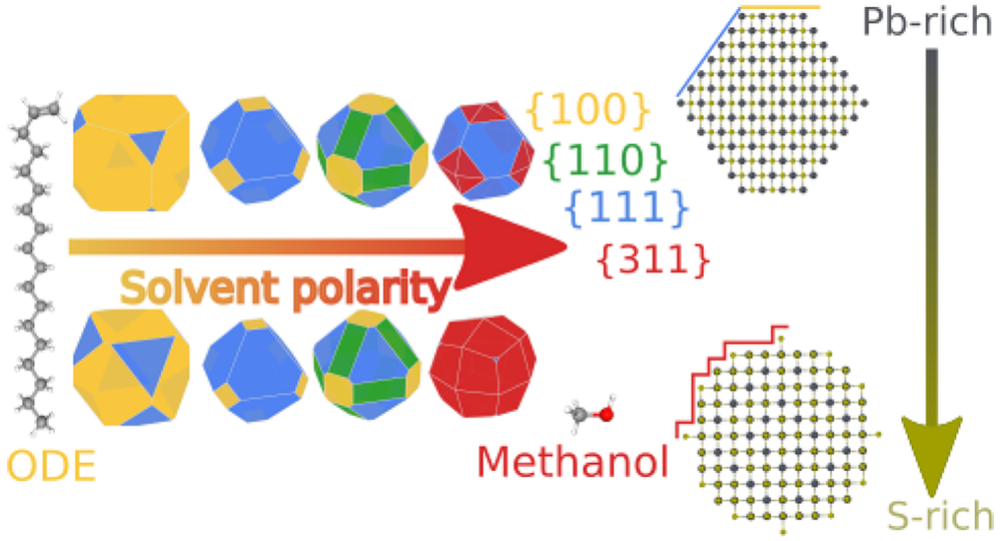

Colloidal nanocrystals (NCs) have shown remarkable promise for optoelectronics, energy harvesting, photonics, and biomedical imaging. In addition to optimizing quantum confinement, the current challenge is to obtain a better understanding of the critical processing steps and their influence on the evolution of structural motifs. Computational simulations and electron microscopy presented in this work show that nanofaceting can occur during nanocrystal synthesis from a Pb-poor environment in a polar solvent. This could explain the curved interfaces and the olivelike-shaped NCs observed experimentally when these conditions are employed. Furthermore, the wettability of the PbS NCs solid film can be further modified via stoichiometry control, which impacts the interface band bending and, therefore, processes such as multiple junction deposition and interparticle epitaxial growth. Our results suggest that nanofaceting in NCs can become an inherent advantage when used to modulate band structures beyond what is traditionally possible in bulk crystals.

Compared with conventional semiconductor manufacturing approaches, solution-processed compound semiconductors combine cost-effective processing, scalable fabrication, and compatibility with flexible substrates, which suggests that they are promising building blocks for next-generation semiconductor technologies.^1,2^ Much progress has been made toward the goal of solution-processed solid films based on multilayer deposition processing and heteroepitaxial growth in the area of colloidal semiconductors technologies, such as the recently commercialized passive-mode photoluminescence colloidal quantum dot semiconductor nanocrystals (NCs) display and lighting products.^3,4^ However, the performance of the corresponding active-mode devices, such as electroluminescence, field effect transistors, and photovoltaic devices, needs further improvement to meet the stringent requirements of industrial applications.^5,6^ The limited understanding of NC processing parameters affecting active-mode device performance remains a challenge in developing further advanced solution-processable technologies.^4−6^

Because of the large surface-area-to-volume ratios and solution-processability of NCs, stoichiometry and solvent effects are considered to be the key internal and external parameters, respectively, that define the unique physicochemical properties of the NCs. To complement the quantum confinement band gap engineering, one can manipulate the electronic and crystal structure properties of NCs by changing the surface bonding states, the solvent, and the overall composition.^7,8^ Carrier mobility can be affected by changing the interparticle dielectric environment and the charge transfer distance.^9^ Additionally, through appropriate surface states and stoichiometry, one can also modulate the electronic trap sites, alter the carrier/exciton lifetimes, and even improve the solubility of NCs.^2,7^

Surface ligands are one of the commonly used stabilizers for preventing oxidation of the NCs surfaces and reducing the agglomeration of nanoparticles.^10^ In particular, mediated by either a polar or nonpolar solvent, the cascaded surface capping ligand exchange can result in atomic-resolution deposition of multiple layers of NCs to form solid films. This process has attracted significant attention in colloidal epitaxial growth for optoelectronic devices.^11−16^ Thus, rationally selected ligands can exchange the “native” ligands through their electrostatic or nucleophilic interactions toward the NCs, and this can provide lattice anchoring sites for new layers of NCs during the deposition process.^17,18^ However, the understanding of the fundamental evolution process of the structural motifs from these nanoscale building blocks is still unclear, and the primary steps and mechanism that determines the solvent wettability of the NCs and the variation in the band structure for different solvents and stoichiometry have been barely explored to date.^19^

Herein, on the basis of density functional theory (DFT) models and electron microscopy studies of a typical rock-salt cubic binary lead sulfide (PbS) model, we reveal that the nanofaceting process is the key step that underpins stoichiometry and solvent effects on the modulation of the surface energy and morphology of individual NCs. The nanofaceting process enables the evolution of local structural motifs, which reflects the changes in their physicochemical and optical–electronic properties. The DFT calculation of the surface energies enables the prediction of the thermodynamically stable morphology. In order to predict the shape of the NCs, the surface energy of the NCs was minimized using a Wulff construction,^20^ as implemented in Wulffpack.^21^ We have taken into consideration not only the low-index {100}, {111}, and {110} facets but also higher-index surfaces, such as the {311} family, that are observed infrequently. The low-index nonpolar {100} family of surfaces is the most stable one in vacuum and results in a near-cubic morphology for the nanocrystal. When solvent polarity is taken into account, polar surfaces become more stable relative to nonpolar ones, and the shape of the nanocrystals changes from a cube to a truncated octahedron.

Once the surface energies have been calculated, we produce a surface phase-diagram, which is shown in Table 1 and Figure 1. The nonpolar {100} surface is shown as a solid black line, while the nonpolar {110} surface is shown as a dotted line. We have considered both terminations of the {111} polar surface: the surface energy of the Pb-terminated facets is shown as a blue dashed-dotted line, and an orange dashed line is used for the S-terminated facet. The (2 × 1) reconstructions (“Wood’s notation”) of the corresponding {111} surfaces are shown as green dashed-dotted lines (Pb-terminated) and red dashed lines (S-terminated). Similarly, {311} polar surfaces are shown as purple dashed-dotted lines (Pb-terminated) and brown dotted lines (S-terminated).

**Table 1:** Calculated Surface Energies in (meV Å^–2^), with Polar Surfaces Labeled by Their Termination (Pb or S) and the (2 × 1) Reconstructions Included in the Case of (111) Surfaces

	Pb-rich			Pb-poor		
surface index	vacuum	ODE	methanol	vacuum	ODE	methanol
(100)	23.7	36.2	34.6	23.7	36.2	34.6
(110)	35.4	35.2	32.1	35.4	35.2	32.1
(111)_Pb_	63	63.2	62.9	98.3	98.8	98.5
(111)_S_	83.4	83.8	83.5	50.4	51	50.6
(111)_Pb_ (2 × 1)	28.4	28.5	25.8	28.4	28.5	25.8
(111)_S_ (2 × 1)	33.8	33.6	30.5	33.8	33.6	30.5
(311)_Pb_	30.5	30.8	30	45	45.2	44.5
(311)_S_	43.6	43.9	42	29.1	29.4	27.5

**Figure 1:**
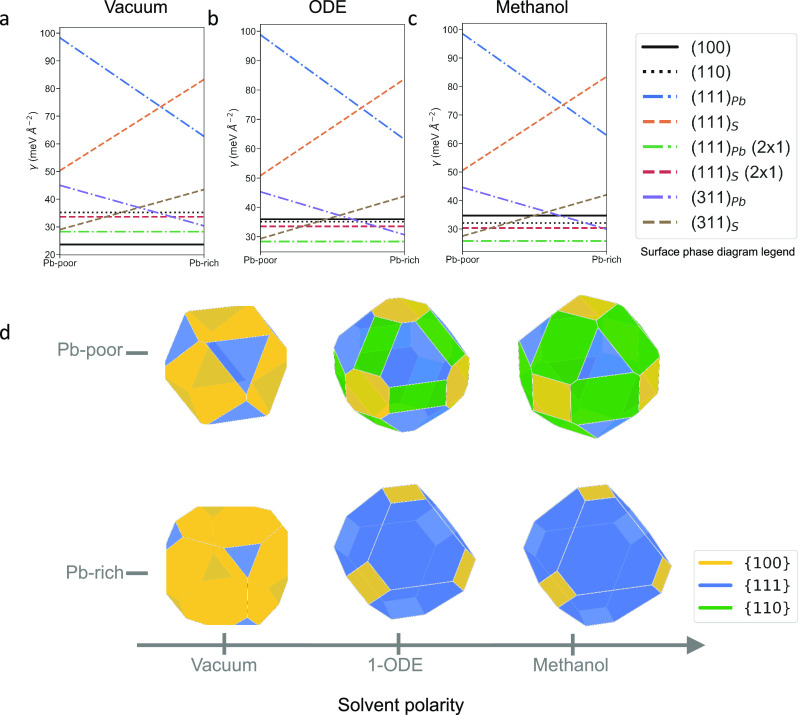
Surface phase diagrams and corresponding surfaces legend of PbS NCs in (a) vacuum, (b) 1-octadecene (ODE), and (c) methanol. (d) The corresponding Wulff constructions of PbS NCs as a function of stoichiometry and solvent polarity. {100} facets are shown in yellow, {111} facets are shown in blue, and {110} facets are shown in green.

In vacuum, surface energies are dominated by the nonpolar {100} facets and the appearance of polar {111} facets, which is similar to previous studies.^22^ In order to capture the solvent effects, we have used an implicit solvation model, as implemented in the VASPsol package.^22,23^ When solvent screening is taken into account (Figure 1b,c), the energy of the nonpolar facets increases, whereas that of the polar facets decreases. These effects increase with the solvent polarity, which validates the materials and device processing protocols that are normally used.^24^

Figure 1d shows detailed local motif variation as a function of stoichiometry. The nonpolar {100} family of surfaces is the most stable in vacuum, which results in a near-cubic morphology for the nanocrystal (Figure 1). In vacuum and Pb-poor conditions, the preferred shape is close to a cube, and Pb-rich conditions result in an increased area of {111} (Pb-terminated) facets. When solvent screening is taken into account (ODE and methanol), the faceting becomes more pronounced. In particular, Pb-rich conditions now result in a truncated octahedron nanocrystal shape that is dominated by {111} (Pb-terminated) facets, while Pb-poor NC models display significant {110} facets that play a role in the experimentally observed self-assembly, which will be further discussed in the microscopy and diffraction analysis.

We prepared different stoichiometric PbS NCs in ODE according to our previous works with slight modifications [Supporting Information (SI), Tables S1 and S2, and Figure S1].^12,25,26^ As shown in Figure 2a, the symmetrically ordered Pb-rich PbS NCs form a superstructure with a standard hexagonal close-packing (*hcp*) arrangement. This is apparent from the transmission electron microscope (TEM) image and the corresponding Fast Fourier transform (FFT) pattern (e.g., intersection angle = 61.42 ± 1.54°). Nonetheless, this periodicity of the superstructure is absent for the S-rich PbS NCs (Figure 2b). Instead, a textured crystal structure with a high packing density is identified, as shown in the selected area electron diffraction (SAED) pattern in Figure 2b (inset image). Powder X-ray diffraction (XRD) analysis (Figure 2c) and high-resolution TEM (HRTEM) analysis (Figure 2d) suggest that these textural crystal features arise from a [110] directional attachment, which breaks the symmetry of the NC arrangement.^27,28^

**Figure 2:**
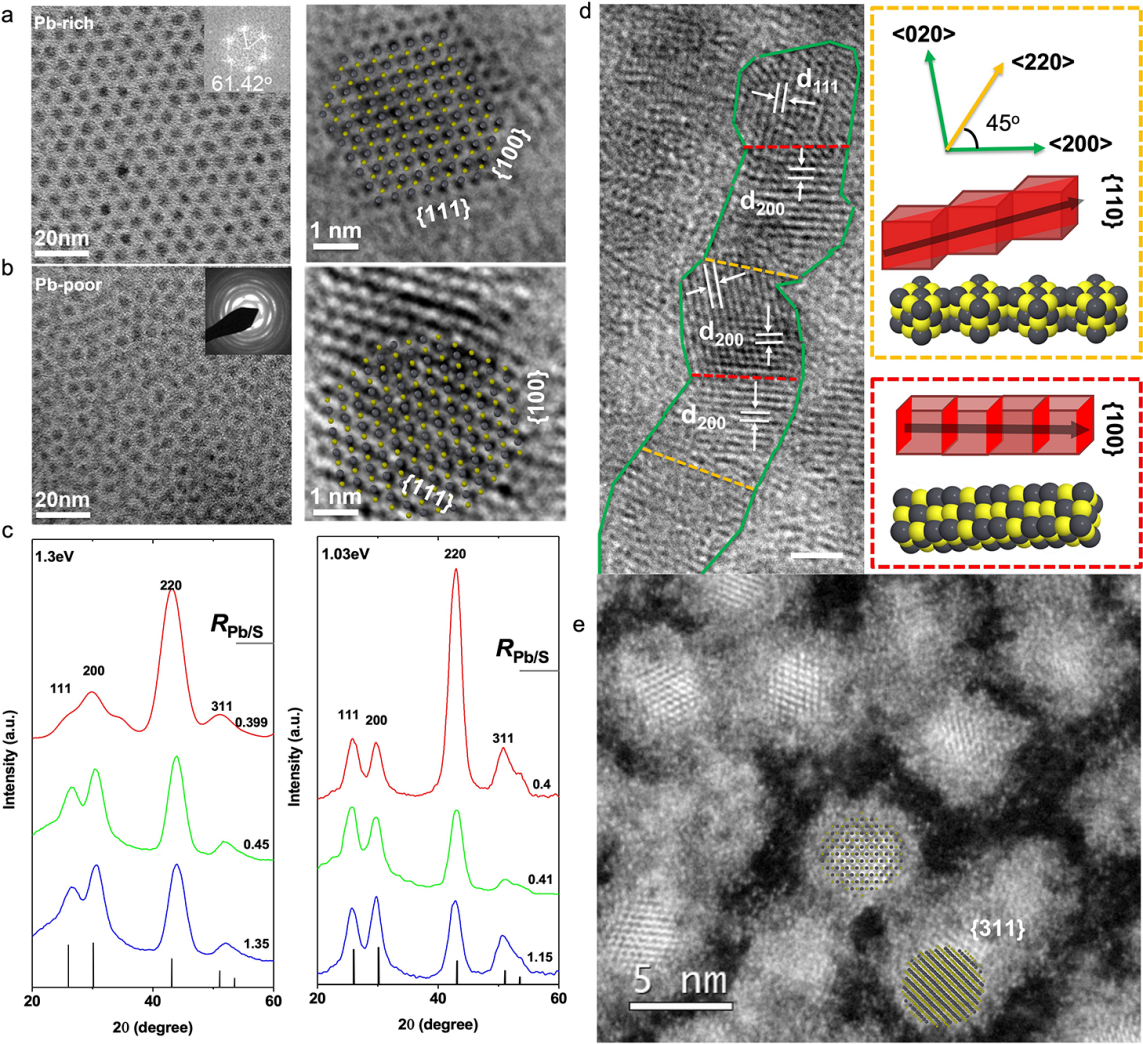
(a,b) TEM (left) and HRTEM (right, view down from [011] zone axis) images of 1.3 eV oleic acid (OA)-capped PbS NCs (PbS@OA) film with Pb-rich (Pb/S = 1.35) and S-rich (Pb/S = 0.39) configurations. Inset images highlight the *hcp* particle packing sequence through generating FFT and the corresponding SAED patterns of the texture structure. HRTEM {100} and {111} cross-grating patterns are also highlighted. (c) XRD patterns of 1.3 and 1.03 eV band gap PbS NC samples with different Pb/S ratios. (d) HRTEM image of S-rich PbS NCs (Pb/S = 0.39). The lattice spacing and bridging orientation were indexed as highlighted in the image, and the scale bar is equal to 1 nm. (e) High-resolution high-angle annular dark-field scanning transmission electron microscopy (HAADF-STEM) images of PbS NCs (Pb/S = 0.39) after methanol washing.

The surface phase diagrams (Figure 1a–c) and Wulff constructions (Figure 1d) show that polar {311} facets can be very low in energy when solvent effects are taken into account. This corroborates previous observations of nanofaceting effects observed in PbSe nanocrystals.^29,30^ The S-terminated {311} facets are also lower in energy in Pb-poor conditions when compared with their Pb-terminated counterparts in Pb-rich conditions. This, in turn, might explain the observed olive shape of PbS nanoparticles synthesized in Pb-poor conditions (HRTEM, Figure 2b) and in a polar solvent (methanol, Figure 2e), as nanofaceting will result in more curved interfaces. A Wulff construction (overlay of atomic models) including these high-index polar facets for a nanocrystal synthesized in Pb-poor conditions and a polar solvent (methanol) is shown in the insets of Figure 2a,b,e (Pb atoms are shown in gray, while S atoms are shown in yellow; see further details in HRTEM simulations in SI, Figures S2 and S3). Alternatively, the elongated particles obtained in Pb-poor conditions (Figure 2d) could be the result of {110} faceting, the formation of dimers, and the resulting reorganization of the NC, which was also previously reported experimentally and studied theoretically.^28,31,32^ It is worth mentioning that {311} facets have been found to be quite rare in the microscopy results (Figure 2e), but they can be difficult to resolve and to differentiate experimentally from the other facets, in particular the {111} facets.

Interestingly, through statistical analysis of HRTEM images (Figure 3a), we found that the Pb-rich NCs (synthesized in ODE) are mainly terminated by {111} planes ({111} population = 56%). However, when we enriched the amount of sulfur in the PbS NCs (synthesized in ODE), the ratio of {200} planes dramatically increased and made the ratio between the {111} and {200} facets change from 8:6 to 8:9 ({200} population = 53%). Because the carboxyl moiety preferentially binds to the Pb atom, a change in the local facet motif of NCs will inevitably affect the population of the surface bonding states and the surface polarity.^33,34^ For all measurements, we have used a cryostat holder to minimize beam damage and reduce the possibility of reorientation, though we cannot completely exclude that NCs may reorient under the electron beam.

**Figure 3:**
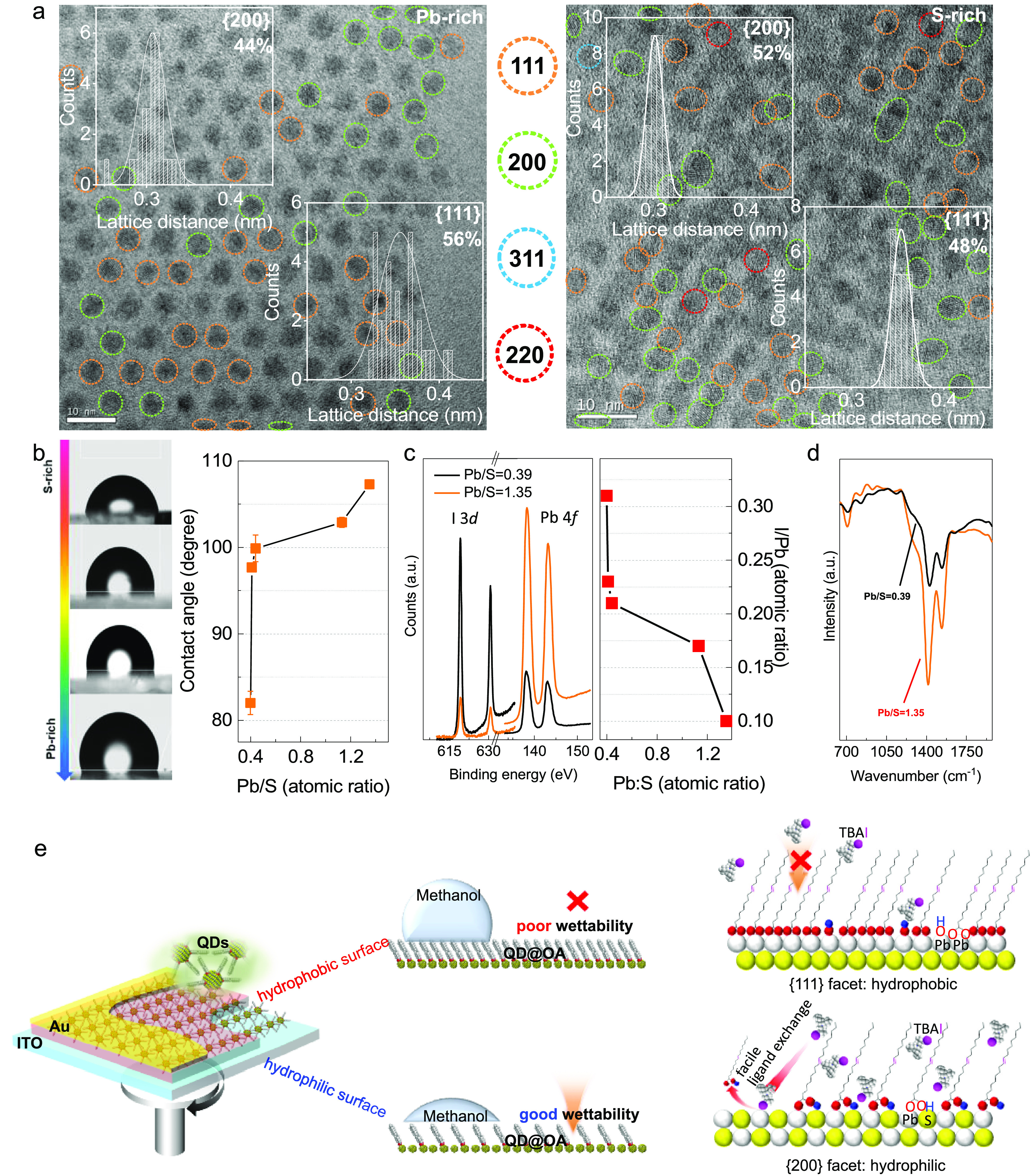
(a) HRTEM images and statistical analysis results (inset) of as-prepared 1.3 eV band gap PbS NCs with Pb/S ratio of 1.35 (left) and 0.39 (right). (b) The contact angle of 1.3 eV band gap PbS@OA NCs with different stoichiometry. (c) XPS spectra (left panel) produced from TBAI-treated off-stoichiometric PbS NCs with a Pb/S ratio equal to 0.39 (black curve) and 1.35 (red curve). The I/Pb atomic ratio as a function of the Pb/S ratio is shown in the right panel. (d) Magnified FTIR spectrum of oleic acid-capped different stoichiometry PbS NCs. (e) A schematic illustration of the solid-state ligand exchange process of the NCs (left panel). The right panel shows the surface bonding configurations of PbS@OA NCs.

As expected, we found that the Pb/S ratio can control the wettability of the PbS@OA films. We show in Figure 3b that the contact angle of the 1.3 eV band gap Pb-rich PbS@OA film is 107.3 ± 4.81°, which is much larger (Δ = ∼25°) than the S-rich PbS@OA film (82.01 ± 2.91°). The solid phase with good wettability can induce a larger volume of the liquid to disperse into the grain boundaries, and it can subsequently enhance ligand exchange efficiency.^35,36^ A methanol-dispersed TBAI ligand solution was selected to replace the long-chain aliphatic ligands on the surface of the NCs in order to investigate the variation of wettability (PbS@TBAI, SI).^25,26,37,38^ The smaller the contact angle is, the better the wettability of the hydrophilic ligand solution (i.e., methanol + TBAI).^35^ Indeed, quantized X-ray photoelectron spectroscopy (XPS) analysis reveals that, after TBAI ligand exchange, the S-rich NCs film has more iodine(I) intercalation than the Pb-rich NCs (Figure 3c). This indicates that an S-rich PbS@OA film can provide a favorable wetting because iodide ions are able to replace more of the carboxyl moieties from the surface of PbS NCs. An important difference between the Pb-rich and Pb-poor NC assemblies is that the Pb-rich conditions favor a more ordered packing arrangement, while Pb-poor conditions result in a disordered assembly of particles. However, for 3D stacking multiple layers of NC films, the NC assemblies seem to be randomly ordered, which may be due to a nonequilibrium dynamical spin coating and ligand exchange process (Figure S4).

In Figure 3d, for those of the same film thickness (SI, Figure S5, Table S3), FTIR and nuclear magnetic resonance (NMR, Figure S6–S7) analysis reveal that Pb-rich PbS NCs films have more OA molecules bonded surface than an S-rich film. This surface deficiency of OA ligands is due to the lack of Pb-terminated sites on the S-rich NCs.^34^ This bonding state variation can subsequently affect the packing arrangement of the NCs.^11^ We believe that the augmented population of {200} planes not only leads to the attachment along the {110} direction of the assembly of the NCs and to the shape changing (e.g., ellipsoidal shape Figure 2b) but it also affects the surface bonding configuration of the as-prepared PbS@OA films.^27,34^ On the basis of theoretical calculations, the binding energy of OA on a PbS NC surface is different between the {111} and {200} planes.^11,34^

As exemplified in Figure 3e, the deprotonated OA and hydroxide moieties are identified to be coordinate-bonded on the {111} planes, which are difficult to eliminate during the solid-state ligand exchange process.^34,39,40^ However, in the case of the {200} planes, the OA was determined to be weakly adsorbed on their surface, which can be easily removed by applying an acidic ligand solution (e.g., methanol + TBAI).^11,26,39,40^ Therefore, through deliberate enrichment of the populations of the {200} planes (i.e., S-rich NCs), we can manipulate the surface wettability of the PbS@OA NCs and, hence, enhance the inorganic (i.e., NCs) and organic (i.e., ligands) interface reaction for improving NC film ligand exchange efficiency and potentially colloidal epitaxy growth.

The NC models for the Pb-rich and Pb-poor NCs of ∼2.4 nm in diameter were generated using the Wulff construction shapes previously obtained and shown in Figure 1. Both NC models are close to a 1:1 Pb:S ratio and have a similar cubic shape that exposes either Pb-terminated or S-terminated polar {111} facets, as shown in Figure 4a,b. In order to passivate these polar facets, counterion ligands were added, namely I atoms were placed on the Pb-terminated {111} facets, following previous work.^41^ In the case of the S-terminated {111} facets, H atoms were used in order to obtain passivation. The electronic structure of these NC models was evaluated, and a partial density of states (DOS) is shown in Figure 4c for the Pb-poor NC model and in Figure 4d for the Pb-rich NC model. Given the structural similarity of the two models, their partial electronic DOS are also quite similar. In both cases, the top of the valence band is dominated by S-p and Pb-s states, while the bottom of the conduction band is dominated by Pb-p states. It is known that DFT calculations can significantly underestimate the band gap.^42^ In order to correct for this, we used the GLLB-SC calculation, including the derivative discontinuity.^43^ The DFT calculation finds that the Pb-poor NC has a bandgap of 1.471 eV compared with 1.604 eV for the Pb-rich. Rather than the absolute values, we focus on the reduction of the band gap (0.13 eV) in Pb-poor conditions, which appears to be caused by the changes in the valence band of the material because of the stoichiometric excess of S. The absolute values of the band gaps of both models are slightly larger than those observed experimentally but are reasonable given the model size (∼2.4 nm) and the approximations made. Most importantly, the DFT estimation, including the derivative discontinuity of the Kohn–Sham potential, is able to reasonably capture the magnitude of the band gap reduction in Pb-poor conditions that was observed experimentally (Figure 4e,f). As shown in Figure 4e,f, red-shifted exciton absorption (0.03 eV) and PLs (0.06 eV) were observed. The band gap reduction trend from the Pb-rich to S-rich also correlates well with the increased packing densities, as observed in Figure 2.^9,44^

**Figure 4:**
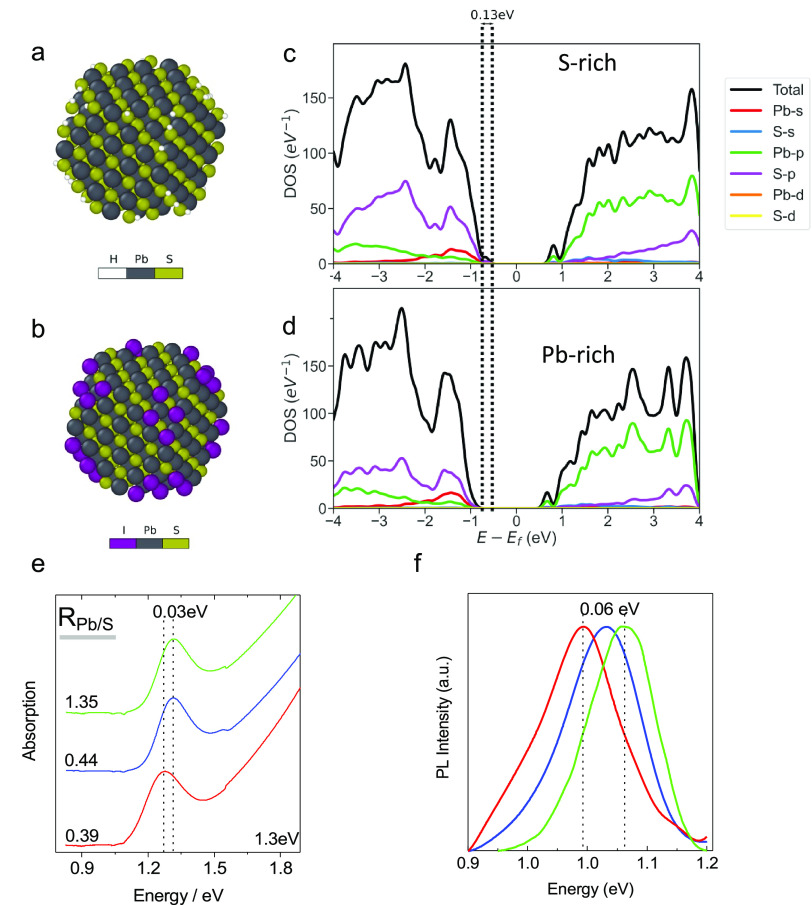
Visualization of a passivated nanocrystal for a PbS NC in S-rich (a) and Pb-rich (b) conditions. Partial density of electronic states of a passivated nanocrystal for a PbS NC in S-rich (c) and Pb-rich (d) conditions. Pb atoms are shown in gray, S atoms are shown in yellow, hydrogen (H) atoms are shown in white, and I atoms are shown in purple. Absorption spectroscopy (e) and photoluminescence (f) analysis results for a typical 1.3 eV bandgap PbS NCs solution in different Pb/S ratios. As highlighted in the figures, the peak position offset is around 0.03 eV (absorption) and 0.06 eV (PL), respectively.

The variation of the Pb/S ratio can also effectively modulate the Fermi levels and DOS in the as-prepared PbS NCs film, which were identified to originate from the charge carrier density alternations.^8^ The variation in the binding energies from PbS NCs of different wettability (e.g., band gap equal to 1.3 eV) was exemplified from ultraviolet photoelectron spectroscopy analysis (UPS, Figure 5a and Figure S8).^12^ As summarized in Figure 5b, when the NC is Pb-rich (hydrophobic), the electron concentration increases, and the Fermi level shifts toward the conduction band (CB). As the NC becomes S-rich (hydrophilic), the hole concentration increases, and the Fermi level drops toward the intrinsic midband gap level. It should be mentioned that a DOS variation (e.g., band edge shifting) with the enrichment of either S or Pb content was also observed from our UPS analysis (Figure S8), which is consistent with previous DFT simulation results.^7,13,27,28,33,40^ This ca. 0.17 eV DOS variation can be attributed to the cation or anion adsorption/desorption on the surface of a NC, which changes the strength of the NC–ligand surface dipole and chemical electronegativity.^7,40^

**Figure 5:**
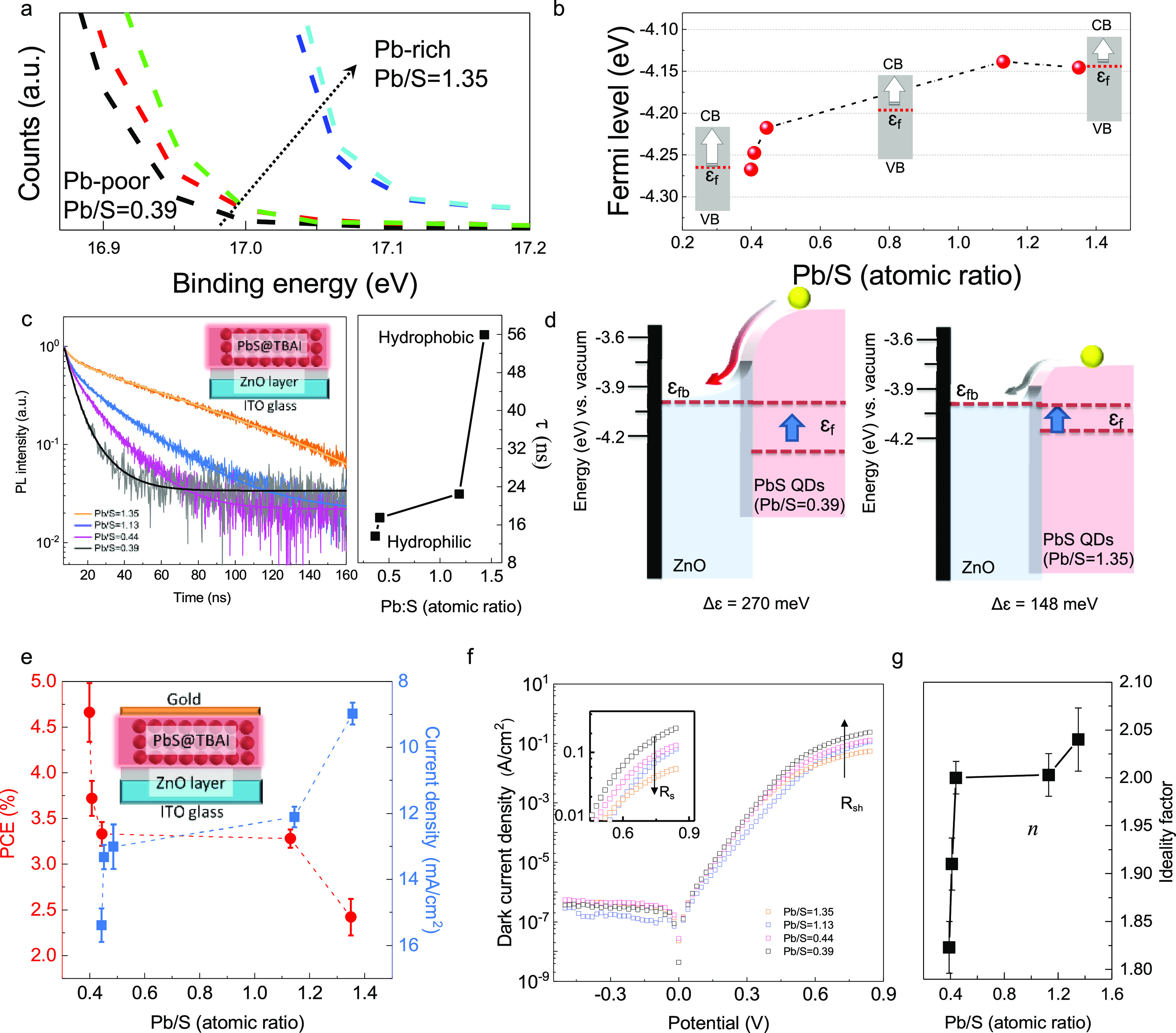
(a) The magnified spectra near the secondary electron cutoff region of the UPS spectra for PbS@TBAI NCs films with different Pb/S ratios. (b) The identified binding energy values (i.e., Fermi levels) as a function of the Pb/S ratio. (c) Time-resolved photoluminescence (TRPL) of the PbS@TBAI NC films different element ratios. Inset image shows a cross-section of the device configuration. (d) The band alignment schematics of the ZnO films (ε_fb_= −4.00) related to 1.3 eV PbS@TBAI in the equilibrium state. The energy levels at the open circuit condition are also provided (red dashed line). Valence band edges of the ZnO scheme are not drawn to scale. (e) Evolution of power conversion efficiency (PCE) (red legend) and *J*_sc_ (blue legend) values as a function of the Pb/S NC ratios. The error bars were generated from standard deviation across nine samples on three different substrates. Inset image shows a cross-section of the photovoltaic device configuration. (f) Semilogarithmic *J*–*V* curves for the dark diode analysis. (g) Calculated ideality factor values (*n*) for different Pb/S ratios. Arrows indicate the increasing direction of *R*_s_ and *R*_sh_.

As shown in Figure 5c, the hydrophilic NC films also display a much shorter PL decay in the time-resolved photoluminescence (TRPL) measurement. For the PL measurement, we employed a ZnO quenching layer, which is the typical electron transport layer in PbS NC-based diode junction devices (Figure 5c, inset). The dynamics of the exciton are complex because they include charge transfer and transport within the ZnO interfaces as the presence of the ZnO layer facilitates carrier extraction, which adds another nonradiative pathway that competes with radiative and trap-assisted recombination.^45,46^ To quantify and compare the photogenerated charge carrier lifetimes, we fit the TRPL decay with a stretched exponential model to obtain lifetime.^47^ The use of this model has been previously suggested to take into account a distribution of monomolecular (e.g., exponential decay) and bimolecular recombination (e.g., biexponential decay) processes, which provide an accepted way of rationalizing complicated exciton dynamics.^47−49^ As shown in Figure 5c, the mean lifetime of a charge carrier reduced from 55.93 ± 0.33 ns to 13.65 ± 0.39 ns as the NCs were modified from being hydrophobic to hydrophilic. A shorter lifetime and decreased PL intensity are indicators of the exciton dissociation having been effectively enhanced.^47^ It proves that the hydrophilic PbS@TBAI NCs can lead to an efficient charge transfer to ZnO NCs by producing a charge-separated state that decays nonradiatively.^45,46^ In Figure 5d, two types of Fermi level (ε_f_) alignment between the flat band potential (ε_fb_) of ZnO and PbS@TBAI are exhibited. In the open-circuit condition, the Fermi level is offset between the ZnO and S-rich or Pb-rich PbS NCs, which were 270 and 148 meV, respectively.

The improved ligand exchange reaction and a steep band bending are beneficial to exciton dissociation and charge collection.^50,51^ The as-prepared NC solar cell (NCSC) power conversion efficiency (PCE) unambiguously shows enhancement because of the S-enrichment. The solar cell structure was similar to our TRPL measurement that employed TBAI as the only ligand (Figure 5c inset, 12 layers of NC@TBAI in total; Figure S5, Table S3) and gold as the anode. As rationalized in Figure 5e, the NCSC PCE performance can be improved more than two times when the NCs are altered from being hydrophobic (PCE = ∼2%) to hydrophilic (PCE = ∼5%). The *J*_sc_ values of the NCSC can be enhanced from 8.98 ± 0.82 mA/cm^2^ to 15.39 ± 0.80 mA/cm^2^, which results from the improved ligand exchange efficiency and the enlarged band bending (Figure S9). Figure 5f,g show the semilog dark current *J*-*V* curves and ideality factor (*n*) as a function of the Pb/S ratio. Benefiting from the large band bending from the S-rich NCs, the charge carrier pathways are largely suppressed under forward bias, which reflects the larger shunt resistance (*R*_sh_), as indicated in Figure 5f. However, the enhanced ligand exchange efficiency also promotes the reduction of Ohmic losses from the series resistance (*R*_s_). The IR drops and minimizes recombination, tunnelling, and inhomogeneity at the junction interface (i.e., decreasing the leakage currents).^52^ Furthermore, the *n* values were estimated by correlating results from both the fitting of the simple diode equation (eq 1)^53,54^ and the solution of the diode equation through conductance derivative methods (eq 2).^55^ In Figure 5g, the *n* values are reduced from 2.04 to 1.82 by enriching the S components in the PbS NCs (Figures S10–S12, and Tables S4–S6), which indicates that the charge recombination across the junction was sufficiently reduced.^38,55,56^
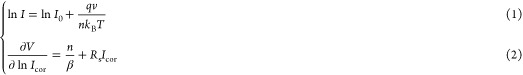


In conclusion, our findings indicate that stoichiometric and solvent control can be used in tandem to stabilize polar surfaces. This nanofaceting process determines the solution-processed NC morphology and the change in optoelectronic properties of devices. The simulation framework deployed here could be further extended to study the stability of NCs further away from a Pb/S ratio of 1:1, as well as the effects of ligand exchange, and work is underway on these topics. Moreover, we believe the nanofaceting is not only limited to PbS NCs but can also apply to other compound semiconductors, such as ternary metal chalcogenides, metal phosphides, and perovskite metal halides.

## Supporting Information Available

Information on QDs and device preparations (Table S1), DFT calculations, composition analysis (Figure S1, Table S2), film structure analysis, electron microscopies experimental details and simulations (HRTEM, FIB, HAADF-STEM, Figures S2–S4; and AFM and DekTak, Figure S5, Table S3), surface chemistry analysis (FTIR, Figure S6; and NMR, Scheme 1, Figure S7), band structure analysis (UPS, Figure S8), XPS and optical property analysis methods (UV–vis-NIR and TRPL), and device characterization (Figures S9–S12, Tables S4–S6) (PDF)

## Notes

Information on the data underpinning the simulation results presented here, including how to access them, can be found in the Cardiff University data catalogue at https://doi.org/10.17035/d.2023.0248150953.

## References

[ref1] Kagan C. R.; Lifshitz E.; Sargent E. H.; Talapin D. V. Building devices from colloidal quantum dots. Science 2016, 353 (6302), aac5523 10.1126/science.aac5523. 27563099

[ref2] Yang Y.; Qin H.; Peng X. Intramolecular Entropy and Size-Dependent Solution Properties of Nanocrystal–Ligands Complexes. Nano Lett. 2016, 16 (4), 2127–2132. 10.1021/acs.nanolett.6b00737. 26923516

[ref3] Li B.; Lu M.; Feng J.; Zhang J.; Smowton P. M.; Sohn J. I.; Park I.-K.; Zhong H.; Hou B. Colloidal quantum dot hybrids: an emerging class of materials for ambient lighting. Journal of Materials Chemistry C 2020, 8 (31), 10676–10695. 10.1039/D0TC01349H.

[ref4] Osypiw A. R. C.; Lee S.; Jung S.-M.; Leoni S.; Smowton P. M.; Hou B.; Kim J. M.; Amaratunga G. A. J. Solution-processed colloidal quantum dots for light emission. Materials Advances 2022, 3, 6773–6790. 10.1039/D2MA00375A.

[ref5] Liu M.; Yazdani N.; Yarema M.; Jansen M.; Wood V.; Sargent E. H. Colloidal quantum dot electronics. Nature Electronics 2021, 4 (8), 548–558. 10.1038/s41928-021-00632-7.

[ref6] Hou B. Colloidal Quantum Dots: The Artificial Building Blocks for New-Generation Photo-Electronics and Photochemistry. Isr. J. Chem. 2019, 59 (8), 637–638. 10.1002/ijch.201900069.

[ref7] Brown P. R.; Kim D.; Lunt R. R.; Zhao N.; Bawendi M. G.; Grossman J. C.; Bulović V. Energy Level Modification in Lead Sulfide Quantum Dot Thin Films through Ligand Exchange. ACS Nano 2014, 8 (6), 5863–5872. 10.1021/nn500897c. 24824726

[ref8] Oh S. J.; Berry N. E.; Choi J.-H.; Gaulding E. A.; Paik T.; Hong S.-H.; Murray C. B.; Kagan C. R. Stoichiometric Control of Lead Chalcogenide Nanocrystal Solids to Enhance Their Electronic and Optoelectronic Device Performance. ACS Nano 2013, 7 (3), 2413–2421. 10.1021/nn3057356. 23368728

[ref9] Liu Y.; Gibbs M.; Puthussery J.; Gaik S.; Ihly R.; Hillhouse H. W.; Law M. Dependence of Carrier Mobility on Nanocrystal Size and Ligand Length in PbSe Nanocrystal Solids. Nano Lett. 2010, 10 (5), 1960–1969. 10.1021/nl101284k. 20405957

[ref10] Tang J.; Kemp K. W.; Hoogland S.; Jeong K. S.; Liu H.; Levina L.; Furukawa M.; Wang X.; Debnath R.; Cha D.; et al. Colloidal-quantum-dot photovoltaics using atomic-ligand passivation. Nat. Mater. 2011, 10 (10), 765–771. 10.1038/nmat3118. 21927006

[ref11] Boles M. A.; Ling D.; Hyeon T.; Talapin D. V. The surface science of nanocrystals. Nat. Mater. 2016, 15 (2), 141–153. 10.1038/nmat4526. 26796733

[ref12] Hou B.; Cho Y.; Kim B. S.; Hong J.; Park J. B.; Ahn S. J.; Sohn J. I.; Cha S.; Kim J. M. Highly Monodispersed PbS Quantum Dots for Outstanding Cascaded-Junction Solar Cells. ACS Energy Letters 2016, 1 (4), 834–839. 10.1021/acsenergylett.6b00294. 28035335 PMC5180466

[ref13] Ip A. H.; Thon S. M.; Hoogland S.; Voznyy O.; Zhitomirsky D.; Debnath R.; Levina L.; Rollny L. R.; Carey G. H.; Fischer A.; et al. Hybrid passivated colloidal quantum dot solids. Nat. Nanotechnol. 2012, 7 (9), 577–582. 10.1038/nnano.2012.127. 22842552

[ref14] Lee S.; Choi M.-J.; Sharma G.; Biondi M.; Chen B.; Baek S.-W.; Najarian A. M.; Vafaie M.; Wicks J.; Sagar L. K.; et al. Orthogonal colloidal quantum dot inks enable efficient multilayer optoelectronic devices. Nat. Commun. 2020, 11 (1), 4814 10.1038/s41467-020-18655-7. 32968078 PMC7511352

[ref15] Balazs D. M.; Dirin D. N.; Fang H.-H.; Protesescu L.; ten Brink G. H.; Kooi B. J.; Kovalenko M. V.; Loi M. A. Counterion-Mediated Ligand Exchange for PbS Colloidal Quantum Dot Superlattices. ACS Nano 2015, 9 (12), 11951–11959. 10.1021/acsnano.5b04547. 26512884 PMC4690194

[ref16] Choi M.-J.; García de Arquer F. P.; Proppe A. H.; Seifitokaldani A.; Choi J.; Kim J.; Baek S.-W.; Liu M.; Sun B.; Biondi M.; et al. Cascade surface modification of colloidal quantum dot inks enables efficient bulk homojunction photovoltaics. Nat. Commun. 2020, 11 (1), 103 10.1038/s41467-019-13437-2. 31900394 PMC6941986

[ref17] Sun B.; Vafaie M.; Levina L.; Wei M.; Dong Y.; Gao Y.; Kung H. T.; Biondi M.; Proppe A. H.; Chen B.; et al. Ligand-Assisted Reconstruction of Colloidal Quantum Dots Decreases Trap State Density. Nano Lett. 2020, 20 (5), 3694–3702. 10.1021/acs.nanolett.0c00638. 32227970

[ref18] Kirmani A. R.; Walters G.; Kim T.; Sargent E. H.; Amassian A. Optimizing Solid-State Ligand Exchange for Colloidal Quantum Dot Optoelectronics: How Much Is Enough?. ACS Applied Energy Materials 2020, 3 (6), 5385–5392. 10.1021/acsaem.0c00389.

[ref19] Xia Y.; Chen W.; Zhang P.; Liu S.; Wang K.; Yang X.; Tang H.; Lian L.; He J.; Liu X.; et al. Facet Control for Trap-State Suppression in Colloidal Quantum Dot Solids. Adv. Funct. Mater. 2020, 30 (22), 2000594 10.1002/adfm.202000594.

[ref20] Wulff G. XXV Zur Frage der Geschwindigkeit des Wachsthums und der Auflösung der Krystallflächen. Zeitschrift für Kristallographie - Crystalline Materials 1901, 34 (1–6), 449–530. 10.1524/zkri.1901.34.1.449.

[ref21] Rahm J. M.; Erhart P. WulffPack: A Python package for Wulff constructions. Journal of Open Source Software 2020, 5 (45), 1944 10.21105/joss.01944.

[ref22] Deringer V. L.; Dronskowski R. Stabilities and Reconstructions of Clean PbS and PbSe Surfaces: DFT Results and the Role of Dispersion Forces. J. Phys. Chem. C 2016, 120 (16), 8813–8820. 10.1021/acs.jpcc.6b02173.

[ref23] Mathew K.; Sundararaman R.; Letchworth-Weaver K.; Arias T. A.; Hennig R. G. Implicit solvation model for density-functional study of nanocrystal surfaces and reaction pathways. J. Chem. Phys. 2014, 140 (8), 084106 10.1063/1.4865107. 24588147

[ref24] Mathew K.; Kolluru V. S. C.; Mula S.; Steinmann S. N.; Hennig R. G. Implicit self-consistent electrolyte model in plane-wave density-functional theory. J. Chem. Phys. 2019, 151 (23), 234101 10.1063/1.5132354. 31864239

[ref25] Hou B.; Kim B.-S.; Lee H. K. H.; Cho Y.; Giraud P.; Liu M.; Zhang J.; Davies M. L.; Durrant J. R.; Tsoi W. C.; et al. Multiphoton Absorption Stimulated Metal Chalcogenide Quantum Dot Solar Cells under Ambient and Concentrated Irradiance. Adv. Funct. Mater. 2020, 30 (39), 2004563 10.1002/adfm.202004563.

[ref26] Hou B.; Cho Y.; Kim B.-S.; Ahn D.; Lee S.; Park J. B.; Lee Y.-W.; Hong J.; Im H.; Morris S. M.; et al. Red green blue emissive lead sulfide quantum dots: heterogeneous synthesis and applications. Journal of Materials Chemistry C 2017, 5 (15), 3692–3698. 10.1039/C7TC00576H. 30009027 PMC6003545

[ref27] Kim D.; Kim D.-H.; Lee J.-H.; Grossman J. C. Impact of Stoichiometry on the Electronic Structure of PbS Quantum Dots. Phys. Rev. Lett. 2013, 110 (19), 196802 10.1103/PhysRevLett.110.196802. 23705733

[ref28] Bertolotti F.; Dirin D. N.; Ibáñez M.; Krumeich F.; Cervellino A.; Frison R.; Voznyy O.; Sargent E. H.; Kovalenko M. V.; Guagliardi A.; et al. Crystal symmetry breaking and vacancies in colloidal lead chalcogenide quantum dots. Nat. Mater. 2016, 15 (9), 987–994. 10.1038/nmat4661. 27295101

[ref29] Cho K.-S.; Talapin D. V.; Gaschler W.; Murray C. B. Designing PbSe Nanowires and Nanorings through Oriented Attachment of Nanoparticles. J. Am. Chem. Soc. 2005, 127 (19), 7140–7147. 10.1021/ja050107s. 15884956

[ref30] Fang C.; van Huis M. A.; Vanmaekelbergh D.; Zandbergen H. W. Energetics of Polar and Nonpolar Facets of PbSe Nanocrystals from Theory and Experiment. ACS Nano 2010, 4 (1), 211–218. 10.1021/nn9013406. 20099911

[ref31] Cui J.; Panfil Y. E.; Koley S.; Shamalia D.; Waiskopf N.; Remennik S.; Popov I.; Oded M.; Banin U. Colloidal quantum dot molecules manifesting quantum coupling at room temperature. Nat. Commun. 2019, 10 (1), 5401 10.1038/s41467-019-13349-1. 31844043 PMC6915722

[ref32] Hou B.; Sohn M.; Lee Y.-W.; Zhang J.; Sohn J. I.; Kim H.; Cha S.; Kim J. M. Chemically encoded self-organized quantum chain supracrystals with exceptional charge and ion transport properties. Nano Energy 2019, 62, 764–771. 10.1016/j.nanoen.2019.05.088.

[ref33] Grisorio R.; Debellis D.; Suranna G. P.; Gigli G.; Giansante C. The Dynamic Organic/Inorganic Interface of Colloidal PbS Quantum Dots. Angew. Chem., Int. Ed. 2016, 55 (23), 6628–6633. 10.1002/anie.201511174. 27038221

[ref34] Zherebetskyy D.; Scheele M.; Zhang Y.; Bronstein N.; Thompson C.; Britt D.; Salmeron M.; Alivisatos P.; Wang L.-W. Hydroxylation of the surface of PbS nanocrystals passivated with oleic acid. Science 2014, 344 (6190), 1380–1384. 10.1126/science.1252727. 24876347

[ref35] Tian J.; Shen T.; Liu X.; Fei C.; Lv L.; Cao G. Enhanced Performance of PbS-quantum-dot-sensitized Solar Cells via Optimizing Precursor Solution and Electrolytes. Sci. Rep. 2016, 6 (1), 23094 10.1038/srep23094. 26975216 PMC4792143

[ref36] Ko D.-K.; Maurano A.; Suh S. K.; Kim D.; Hwang G. W.; Grossman J. C.; Bulović V.; Bawendi M. G. Photovoltaic Performance of PbS Quantum Dots Treated with Metal Salts. ACS Nano 2016, 10 (3), 3382–3388. 10.1021/acsnano.5b07186. 26909739

[ref37] Liu M.; Voznyy O.; Sabatini R.; García de Arquer F. P.; Munir R.; Balawi A. H.; Lan X.; Fan F.; Walters G.; Kirmani Ahmad R.; et al. Hybrid organic–inorganic inks flatten the energy landscape in colloidal quantum dot solids. Nat. Mater. 2017, 16 (2), 258–263. 10.1038/nmat4800. 27842072

[ref38] Chuang C.-H. M.; Brown P. R.; Bulović V.; Bawendi M. G. Improved performance and stability in quantum dot solar cells through band alignment engineering. Nat. Mater. 2014, 13 (8), 796–801. 10.1038/nmat3984. 24859641 PMC4110173

[ref39] Weidman M. C.; Beck M. E.; Hoffman R. S.; Prins F.; Tisdale W. A. Monodisperse, Air-Stable PbS Nanocrystals via Precursor Stoichiometry Control. ACS Nano 2014, 8 (6), 6363–6371. 10.1021/nn5018654. 24840645

[ref40] Cao Y.; Stavrinadis A.; Lasanta T.; So D.; Konstantatos G. The role of surface passivation for efficient and photostable PbS quantum dot solar cells. Nature Energy 2016, 1 (4), 16035 10.1038/nenergy.2016.35.

[ref41] Liu Y.; Kim D.; Morris O. P.; Zhitomirsky D.; Grossman J. C. Origins of the Stokes Shift in PbS Quantum Dots: Impact of Polydispersity, Ligands, and Defects. ACS Nano 2018, 12 (3), 2838–2845. 10.1021/acsnano.8b00132. 29513986

[ref42] Perdew J. P. Density Functional Theory and the Band Gap Problem. Int. J. Quantum Chem. 1985, 28 (S19), 497–523. 10.1002/qua.560280846.

[ref43] Kuisma M.; Ojanen J.; Enkovaara J.; Rantala T. T. Kohn-Sham Potential with Discontinuity for Band Gap Materials. Phys. Rev. B 2010, 82 (11), 115106 10.1103/PhysRevB.82.115106.

[ref44] Greenham N. C.; Peng X.; Alivisatos A. P. Charge separation and transport in conjugated-polymer/semiconductor-nanocrystal composites studied by photoluminescence quenching and photoconductivity. Phys. Rev. B 1996, 54 (24), 17628–17637. 10.1103/PhysRevB.54.17628. 9985889

[ref45] Koole R.; Liljeroth P.; de Mello Donegá C.; Vanmaekelbergh D.; Meijerink A. Electronic Coupling and Exciton Energy Transfer in CdTe Quantum-Dot Molecules. J. Am. Chem. Soc. 2006, 128 (32), 10436–10441. 10.1021/ja061608w. 16895408

[ref46] Bai G.; Zou Y.; Li Y.; Cai L.; Chen B.; Zang J.; Hong Z.; Chen J.; Chen Z.; Duhm S.; et al. Revealing a Zinc Oxide/Perovskite Luminescence Quenching Mechanism Targeting Low-Roll-off Light-Emitting Diodes. J. Phys. Chem. Lett. 2022, 13 (13), 3121–3129. 10.1021/acs.jpclett.2c00564. 35357156

[ref47] Habisreutinger S. N.; Wenger B.; Snaith H. J.; Nicholas R. J. Dopant-Free Planar n–i–p Perovskite Solar Cells with Steady-State Efficiencies Exceeding 18. ACS Energy Letters 2017, 2 (3), 622–628. 10.1021/acsenergylett.7b00028.

[ref48] Noel N. K.; Habisreutinger S. N.; Wenger B.; Klug M. T.; Hörantner M. T.; Johnston M. B.; Nicholas R. J.; Moore D. T.; Snaith H. J. A low viscosity, low boiling point, clean solvent system for the rapid crystallisation of highly specular perovskite films. Energy Environ. Sci. 2017, 10 (1), 145–152. 10.1039/C6EE02373H.

[ref49] Stranks S. D.; Eperon G. E.; Grancini G.; Menelaou C.; Alcocer M. J. P.; Leijtens T.; Herz L. M.; Petrozza A.; Snaith H. J. Electron-Hole Diffusion Lengths Exceeding 1 Micrometer in an Organometal Trihalide Perovskite Absorber. Science 2013, 342 (6156), 341–344. 10.1126/science.1243982. 24136964

[ref50] Speirs M. J.; Dirin D. N.; Abdu-Aguye M.; Balazs D. M.; Kovalenko M. V.; Loi M. A. Temperature dependent behaviour of lead sulfide quantum dot solar cells and films. Energy Environ. Sci. 2016, 9 (9), 2916–2924. 10.1039/C6EE01577H.

[ref51] Bozyigit D.; Lin W. M. M.; Yazdani N.; Yarema O.; Wood V. A quantitative model for charge carrier transport, trapping and recombination in nanocrystal-based solar cells. Nat. Commun. 2015, 6 (1), 6180 10.1038/ncomms7180. 25625647 PMC4317500

[ref52] Clifford J. P.; Johnston K. W.; Levina L.; Sargent E. H. Schottky barriers to colloidal quantum dot films. Appl. Phys. Lett. 2007, 91 (25), 253117 10.1063/1.2823582.

[ref53] Sze S. M.; Ng K. K. Physics of Semiconductor Devices; Wiley, 2006.

[ref54] Werner J. H. Schottky barrier and pn-junctionI/V plots — Small signal evaluation. Appl. Phys. A: Mater. Sci. Process. 1988, 47 (3), 291–300. 10.1007/BF00615935.

[ref55] Chuang C.-H. M.; Maurano A.; Brandt R. E.; Hwang G. W.; Jean J.; Buonassisi T.; Bulović V.; Bawendi M. G. Open-Circuit Voltage Deficit, Radiative Sub-Bandgap States, and Prospects in Quantum Dot Solar Cells. Nano Lett. 2015, 15 (5), 3286–3294. 10.1021/acs.nanolett.5b00513. 25927871 PMC4754979

[ref56] In the equations, *I*_0_ is the saturation current density, *I*_cor_ is the corrected current after subtracting the shunt current, *q* is the electronic charge, *V* is the potential drop across the junction, *n* is the ideality factor, *k*_B_ is Boltzmann’s constant, and *T* is the temperature. The *n* values reported here are average values from the two approaches (see further details in the Supporting Information).

