# Supplementary material for: Evolution of Local Structural Motifs in Colloidal Quantum Dot Semiconductor Nanocrystals Leading to Nanofaceting
Source: Nano Lett. 2023 Mar 13;23(6):2277–86. doi: 10.1021/acs.nanolett.2c04851 (PMC10037336; doi:10.1021/acs.nanolett.2c04851)
Supplement: Supplementary file 1 — nl2c04851_si_001.pdf [file nl2c04851_si_001.pdf]

Supporting Information

Evolution of Local Structural Motifs in Colloidal Quantum Dot Semiconductor Nanocrystals Leading to Nanofaceting

Bo Hou[*,a,§], Felix Cosmin Mocanu[*,b,§], Yuljae Cho[c,d], Jongchul Lim[e], Jiangtao Feng[f], Jingchao Zhang[g], John Hong[c,h], Sangyeon Pak[c,i], Jong Bae Park[c], Young-Woo Lee[c], Juwon Lee[c], Byung-Sung Kim[c], Stephen M. Morris[c], Jung Inn Sohn[j,*], SeungNam Cha[k,*] and Jong Min Kim[l]

[a]School of Physics and Astronomy, Cardiff University, Queen's Building, The Parade, Wales, CF24 3AA, United Kingdom. Email: Houb6@cardiff.ac.uk

[b]Laboratoire de Physique de l'École Normale Supérieure, ENS, Université PSL, CNRS, Sorbonne Université, Université de Paris, 75005 Paris, France. Email: felix-cosmin.mocanu@phys.ens.fr

[c]Department of Engineering Science, University of Oxford, Parks Road, Oxford OX1 3PJ, United Kingdom.

[d]University of Michigan–Shanghai Jiao Tong University Joint Institute, Shanghai Jiao Tong University, 800 Dong Chuan Road, Minghang District, Shanghai 200240, China.

[e]Graduate school of energy science and technology, Chungnam National University, Daejeon, 34134, Republic of Korea.

[f]Department of Environmental Science & Engineering, School of Energy and Power Engineering, Xi'an Jiaotong University, Xi'an, 710049, China.

[g]Microsoft Corporation, Redmond, WA 98052, USA

[h]School of Materials Science and Engineering, Kookmin University, Seoul 02707, Republic of Korea

[i]School of Electronic and Electrical Engineering, Hongik University, Seoul 04066, Republic of Korea

[j]Division of Physics and Semiconductor Science, Dongguk University-Seoul, Seoul 04620, Republic of Korea. Email: junginn.sohn@dongguk.edu

[k]Department of Physics, Sungkyunkwan University, Suwon, Gyeonggi-do 16419, Republic of Korea. Email: chasn@skku.edu

[l]Department of Engineering, Electrical Engineering Division, 9 JJ Thomson Avenue, University of Cambridge, Cambridge, CB3 0FA, United Kingdom

§ B. H. and F. C. M. contributed equally to the work.

SI. Sample Preparation:

Synthesis of PbS NCs

The synthesis of different stoichiometric PbS NCs was analogous to our previous report.[1,2] Briefly, for 1.3 eV band gap PbS NCs, a Pb precursor was prepared in a two-neck flask which was loaded with 0.47 g (2.106 mmol) of lead oxide (PbO), 2.24 mL (6.318 mmol) of oleic acid (OA) (PbO:OA = 1:3) and 12.7 mL of octadecene (ODE). The solution was degassed at 100 °C in vacuum for two hours until a clear solution was formed. Meanwhile, a different amount (see Table S1) of hexamethyldisilathiane (bis (trimethylsilyl) sulfide (TMS) was dissolved into 6.4 ml of ODE in a two-neck flask under vacuum. After 2h, the TMS precursor solution was switched to an Ar atmosphere at room temperature and the Pb precursor flask was filled with Ar and heated to 130 °C. The sulfur precursor was swiftly injected into the Pb reaction flask, and the reaction flask was subsequently cooled to room temperature by an ice bath. The raw reaction solution was purified by adding a mixture of 5 mL of hexane and 25 mL of acetone followed by centrifugation at 8000 rpm. Then, the nanocrystals were washed twice by dissolving them in hexane and precipitating with ethanol (1/10, v/v). Finally, PbS NCs was dispersed into toluene with a weight concentration of 50 mg/mL for device fabrication and characterization (see Table S1 for other stoichiometric NC synthesis).

Table S1. A summary of the synthesis conditions for 1.3 eV PbS NCs with different Pb/S atomic ratios.

Band gap	PbO (g)	OA (mL)	TMS (uL)	Temperature (°C)	Pb/S (atomic ratio)
1.3eV	0.47	2.23	190	130	1.35
	0.47	2.23	200	130	1.13
	0.47	2.23	210	130	0.43
	0.47	2.23	210~220	130	0.41
	0.47	2.23	220~230	130	0.39

Synthesis and purification of ZnO NCs.

The fabrication of ZnO NCs was similar to our previous report.[3] 0.9788g zinc acetate dihydrate was dissolved in 42 mL of methanol and the solution was heated to 60 °C under air. 0.469g of KOH was dissolved in 22 mL of methanol and added into the zinc acetate reaction flask over a period of 15 min in a drop-wise fashion. The total reaction time was fixed at 90 min by quenched the reaction through cooling down to room temperature. The ZnO NCs were purified three times through centrifuging and dispersing in methanol. Finally, the ZnO NCs were dissolved in chloroform at 50 mg/mL.

Nanocrystal Solar cells (NCSCs) fabrication. A solution of ZnO nanoparticles (50 mg/mL) was spin coated onto ITO substrates at 2000 rpm for 30 seconds followed by thermal annealing at 250 °C for 10 min. A 1.3 eV PbS solution (50 mg/mL) was spin casted onto the substrate at 2000 rpm for 15 seconds. A methanol (MeOH) dispersed tetrabutylammonium iodide (TBAI) solution (10 mg/mL) was applied to the PbS NC layer for 30 seconds, then spin coated at 2000 rpm for another 30 second, followed by two rinse-spin steps with MeOH (Figure S3). For the TBAI/EDT structure[1,2], a 0.02 vol% EDT in acetonitrile solution was applied to the film for 30 seconds, followed by an acetonitrile rinsing step, which was conducted twice (Figure S12). Au contact pins were thermally evaporated by employing an EDWARD thermal evaporator. The solar cells active area of the solar cells was defined by a 0.0134 cm^2 mask.

Density-functional theory calculations

Plane-wave DFT calculations were carried out using the Vienna ab initio Simulation Package (VASP 5.4.4).[3] Projector augmented-wave (PAW) sets were chosen according to Materials Project guidelines.[4] A maximum plane wave cutoff of 600 eV was used together with a k-point spacing of 0.015 $Å^{-1}$ to ensure the convergence of the calculations. In the case of slab calculations, a vacuum separation of 15 Å was used along the *z*-direction. The self-consistency cycles were halted when the total energy was converged to within $< 10^{-6}$ eV/cell and the

structures were optimized until forces on the atoms were < 10^{-3} eV $Å^{-1}$. We have treated exchange and correlation at the generalized gradient approximation (GGA) level using the PBEsol functional. This approximation was found to provide an accurate description of the structures and mechanical properties of solids and surfaces at a reasonable cost.[5] In order to carry out the larger calculations of NC models and different surface/ligand interfaces we have instead used the GPAW code[6] with an atomic orbital basis of "dzp" quality, PAW sets and the same PBEsol functional.[7] An improved estimate of the quantum-dot band gap was obtained via the GLLB-SC approximation.[8]

The surface energy γ of a relaxed and pristine slab of cubic PbS is given by:

$$\gamma_{pristine} = \frac{E_{slab} - N_{Pb} \cdot \mu_{Pb} - N_S \cdot \mu_S}{2A}$$

where E_{slab} is the energy of the slab. N_{Pb} and N_S are the number of Pb and S atoms respectively while μ_{Pb} and μ_S are the corresponding chemical potentials. Under the conditions of thermodynamic equilibrium, the surface energy can be rewritten to depend only on the chemical potential of one of the species (in this case Pb) and the energy of the relevant bulk phase E_{bulk} (in this case cubic PbS) in the following way:

$$\gamma_{pristine} = \frac{E_{slab} - E_{PbS}^{bulk} - (N_{Pb} - N_s) \cdot \mu_{Pb}}{2A}$$

The surface Gibbs free-energy of the NCs is obtained as the sum of the surface energies γ_i of individual facets multiplied by the respective area A_i giving:

$$\Delta G = \sum_i \gamma_i A_i$$

SII. Material Characterization:

Composition Analysis

X-ray photoelectron spectroscopy (XPS) analysis was performed on a Thermo Scientific K-Alpha instrument equipped with a micro-focused monochromatic Al X-ray source. The source was operated at 12 keV and a 400-micron spot size was used. The analyzer operates at a constant analyzer energy (CAE) 200 eV for survey scans and 50 eV for detailed scans. Charge neutralization was applied using a combined low energy/ion flood source. The data acquisition and analysis were performed with Thermo Scientific Advantage software. Peak fitting (Lorentzian / Gaussian (L/G) 30%) was applied following removal of a Smart background. Normalized atomic percentages were determined from peak areas of the elemental main peaks detected on the survey scan following background subtraction and application of Thermosensitivity factors.

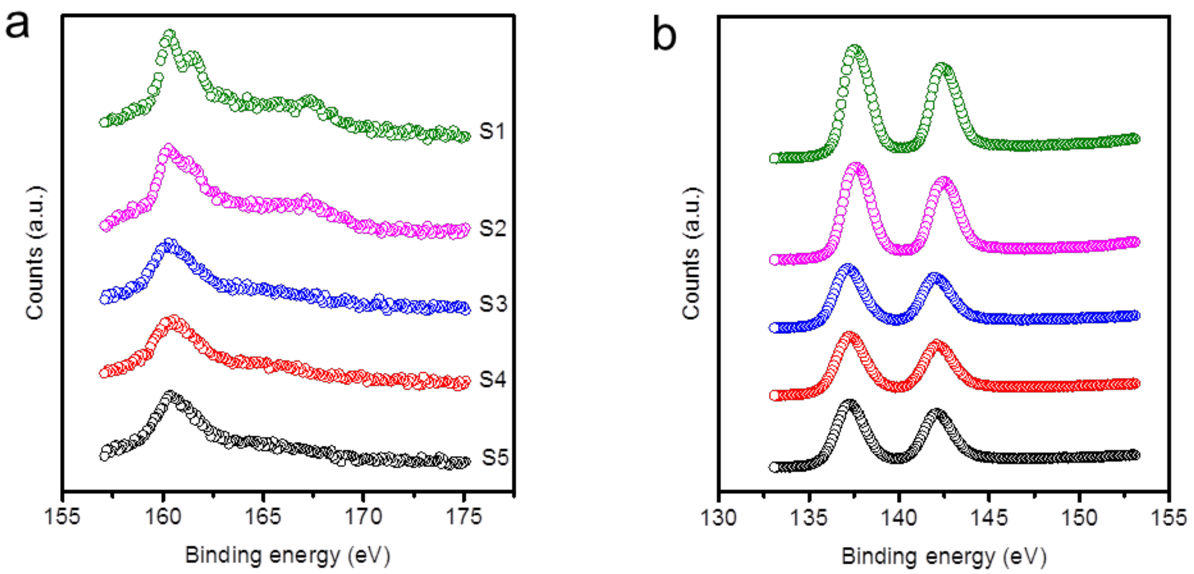

Figure S1. XPS spectra of the 1.3 eV PbS NCs film on the top of ITO slides. a) and b) examples of the S2p and Pb4f spectra from different stoichiometry PbS NCs, respectively.

Table S2. Quantitated XPS analysis results of the atomic ratio between Pb and S from Figure S1.

Sample	Area (N) TPP-2M (Pb4f)	Area (N) TPP-2M (S2p)	Pb atomic %	S atomic %	Pb/S ratio
S1	1071.25	793.22	5.58	4.13	1.35
S2	916.04	811.34	4.7	4.16	1.13
S3	638.86	1472.44	4.44	10.24	0.43
S4	648.22	1618.4	4.35	10.86	0.41
S5	682.54	1695.7	4.67	11.59	0.39

Transmission microscopy (TEM), high resolution TEM, high annular dark-field scanning transmission electron microscope (HAADF-STEM), Focus ion beam (FIB), selected-area electron diffraction (SAED) and X-ray diffraction (XRD) characterizations.

The TEM and HRTEM images were taken using a JEOL-3000F at 300 kV and the SAED analysis was performed at 300 kV with a camera length of 255.8 mm. A cryostat holder was employed for all the measurements, in order to minimise the beam damage and possibility of reorientation. XRD analysis was carried out using a Bruker D8 ADVANCE Diffractometer employing copper kα radiation (λ = 0.15406nm). The sample was continuously spun during data collection and scanned using a step size of 0.02° (2θ) between the range of 10°-90°. Atomic-resolution aberration-corrected high annular dark-field scanning transmission electron microscope (HAADF-STEM) was conducted on a JEOL ARM-200F at 200 KV (cold field emission source Cs probe corrected). Focus ion beam (FIB) - a FEI Helios SEM/FIB system is used for sample milling in order to prepare cross-section HAADF-STEM and HRTEM specimen.

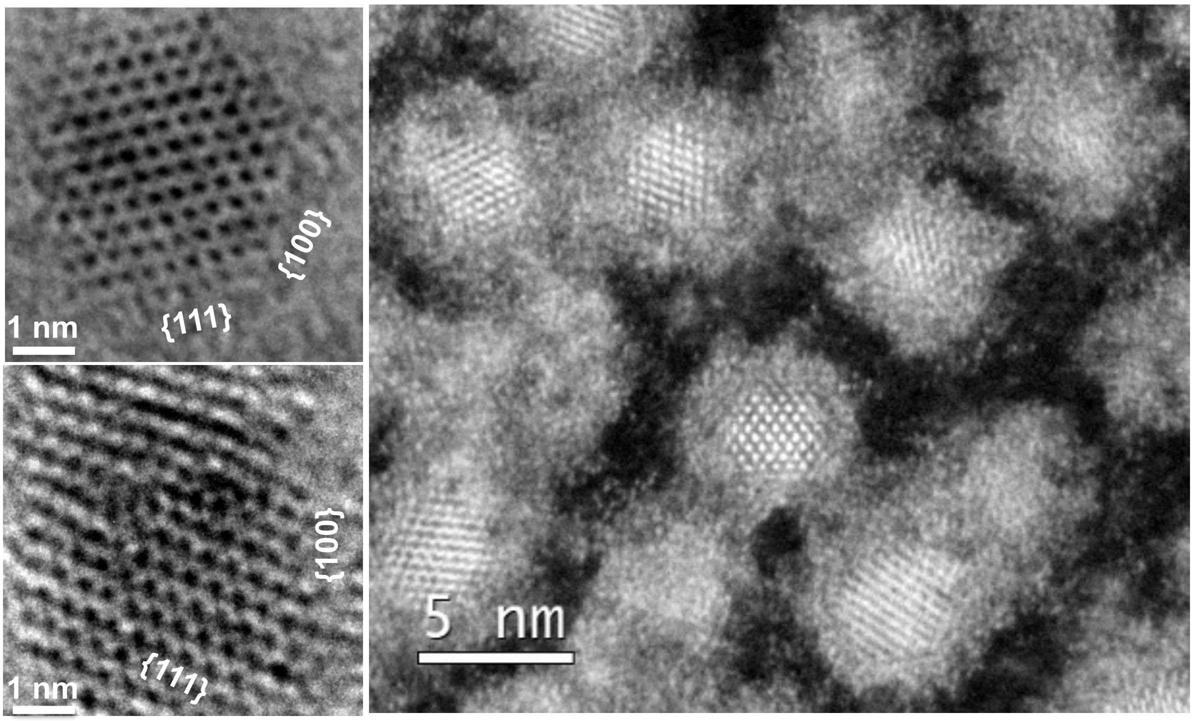

Figure S2. Original HRTEM and HAADF-STEM images from Figure 2 without overlay of atomic models.

Electron microscopy image simulation

To guide the interpretation of HRTEM images (Figure 2 and Figure 3) we have carried out image simulation using the abTEM code.[15,16] We have used the PRISM algorithm to speed-up the calculation.[17] The simulated HRTEM image results are shown in Figure S3 where they can be compared with our atomic NC models obtained from the Wulff construction in the case of Pb-poor conditions in a polar solvent (MeOH).

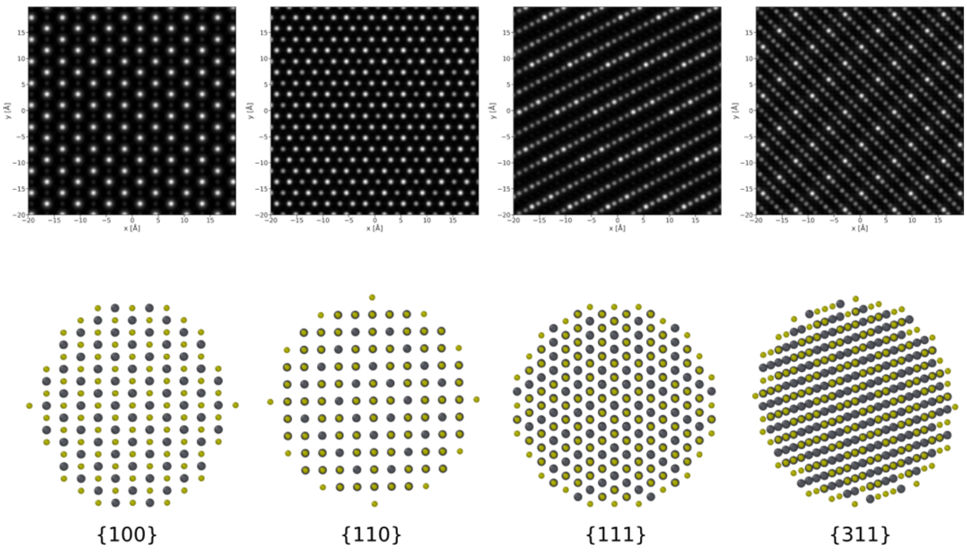

Fig S3.TEM image simulations of (from left to right): {100}, {110}, {111} and {311} facets of the PbS cubic crystal. Corresponding facets shown for an atomic model of a PbS nanocrystal obtained via the Wulff construction for Pb-poor conditions and MeOH solvation.

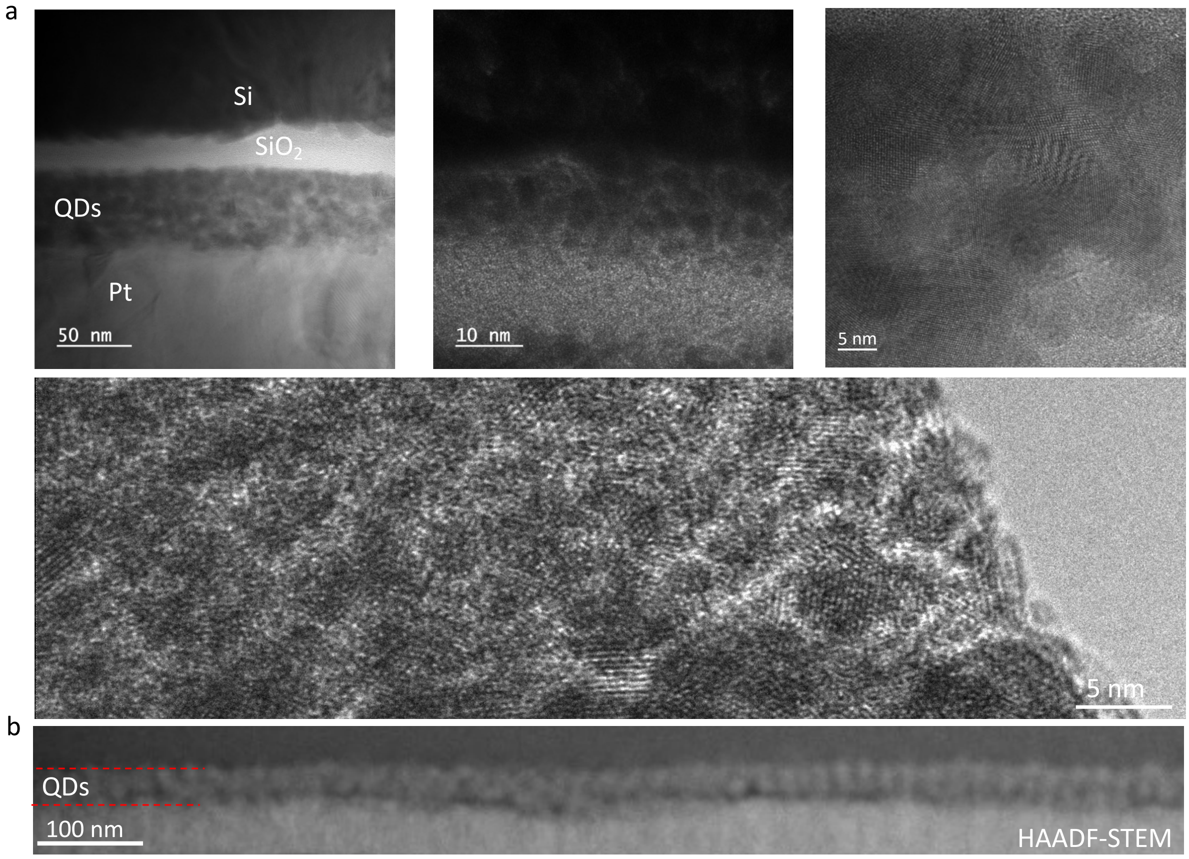

Figure S4. 3D stacking structure analysis using FIB prepared lamella sample. The QD thin films were prepared by 3 layers of 1.3 eV PbS QDs (Pb/S=0.39) on Si wafer (after TBAI ligand exchange). a) HRTEM images, b) HAADF-STEM image.

NC Film Structure and Physical Property Analysis

The film thickness of as-prepared PbS NC films was evaluated using both atomic force microscopy (AFM, Veeco Dimension 3100) and a Veeco DekTak 6M stylus profiler. The absolute change in thickness as a function of stoichiometric (Table S1 and S2) was found to be around 3 nm, which can be neglected the systematic measurement error (~5 nm) is taken into account. Therefore, the film thickness largely depended on the number of deposited layers.

Contact angle measurements were performed according to the procedure described below. A straight needle was used for the manual deposition of the drop for contact angle measurements. The

syringe and the sample were temperature-controlled using a circulating bath. The light source was an integrating sphere type, which gave homogeneous illumination in all the directions. This design gave a uniform illumination of the drop, and hence homogeneous contrast of the drop contour with a light background. A telecentric lens system was used so that the magnification did not depend on the distance between the camera and the drop. Therefore, the optical calibration only needed to be completed when the optical system had been modified and not between samples. Also, this type of lens only caused a small optical distortion (for which the software applied a correction). The CCD camera with 320x256 pixels collected 25 images per second. The data was acquired and the instrument was controlled by a computer equipped with a frame grabber, an A/D data acquisition card and a motor control card. The built-in software enabled the user to both set the instrument (ensuring the needle was vertical, volumetric calibration of the syringes, optical calibration, etc) and obtain data (images, contact angles, temperature, etc). The conditions were set at 20 degrees centigrade and ambient pressure.

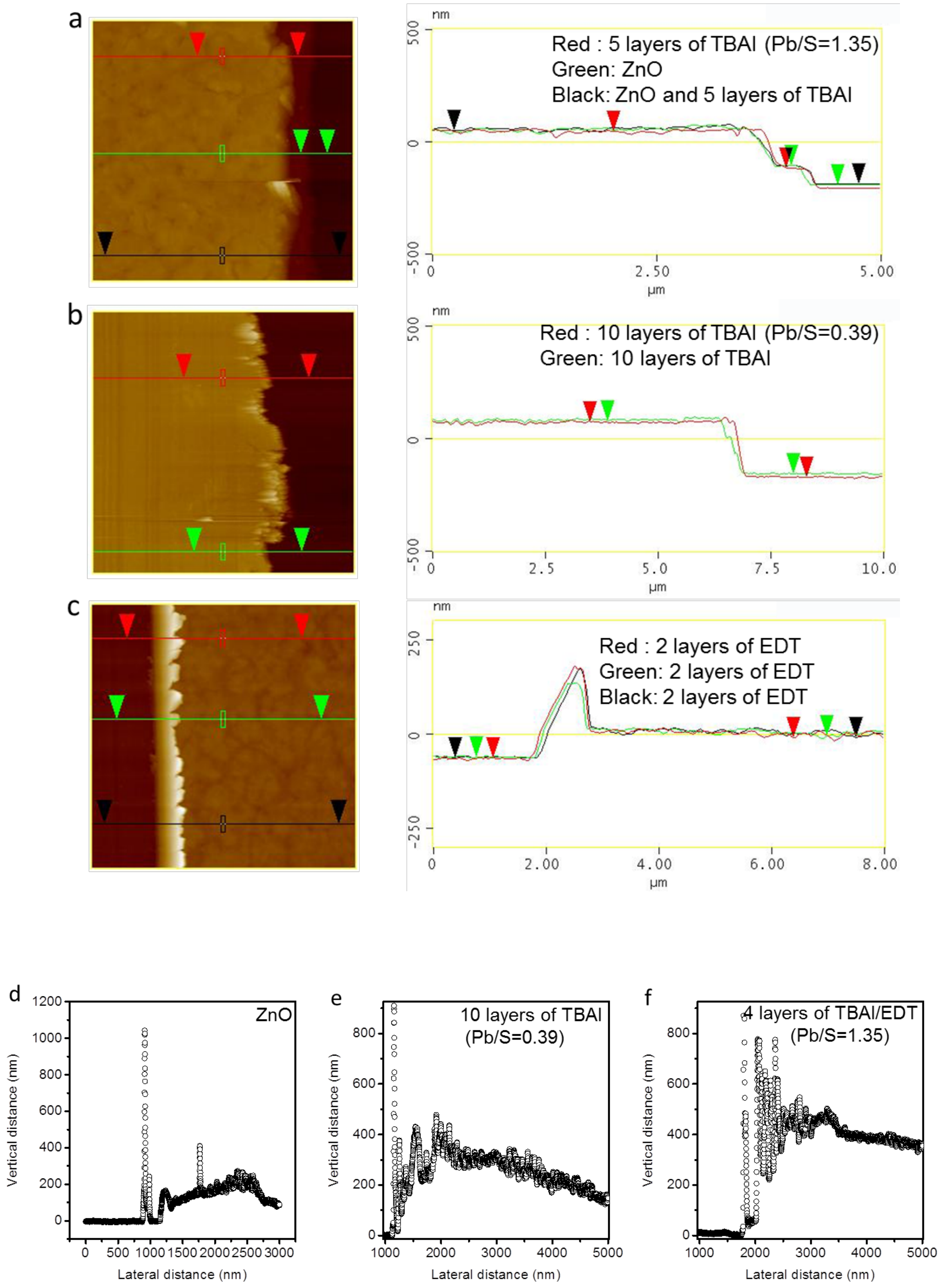

Figure S5. NC layer thickness analysis using AFM a-c) and a DekTak profiler d-f). The layer thickness data is listed in Table S4.

Table S3. Film thickness results extracted from an AFM and a DekTak profiler.

Sample	AFM (nm)	DekTak (nm)	Average (nm)
TBAI	27.9 ± 3.8	25.25 ±7.1	26.6 ± 5.5
ZnO	92.9 ± 6.0	113.6 ± 38	103.25 ±22.1
EDT	27.4 ± 0.5	39.8 ±1.7	33.6±1.1

NC Surface Chemistry Analysis

Three layers of PbS NCs on pre-cleaned glass substrates were employed for FTIR analysis. Fourier transform infrared (FTIR) spectra were taken by a Varian Excalibur FTS 3500 spectrometer. 1D ^{1}H nuclear magnetic resonance spectra were recorded on a 400 MHz Bruker AVH400 spectrometer. Chemical shifts were quoted in parts per million (ppm) and referenced to $SiMe_4$ (^{1}H NMR 0 ppm) and $CDCl_3$ (^{13}C NMR 77.16 ppm). An identical amount of PbS NCs (15mg) were dispersed into $CDCl_3$ for the NMR analysis.

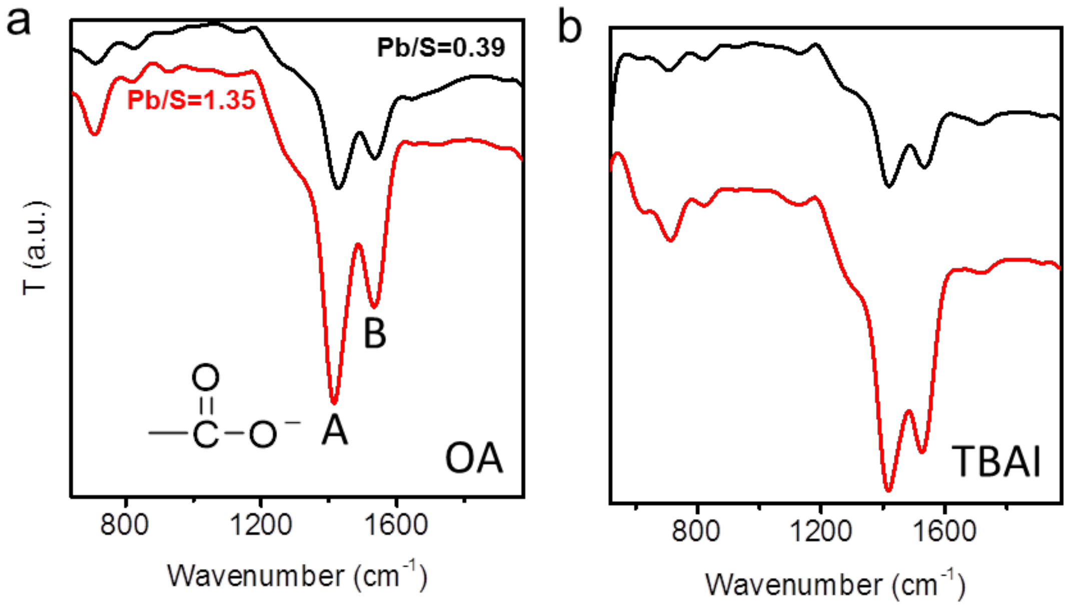

A 1417cm^{-1} carboxylate ion carbonyl symmetrical stretching

B 1534cm^{-1} carboxylate ion carbonyl anti-symmetrical stretching

Figure S6. FTIR analysis of PbS NCs before a) and after TBAI ligand exchange b). The Pb/S ratio and PbS surface functional molecules are highlighted in the image.

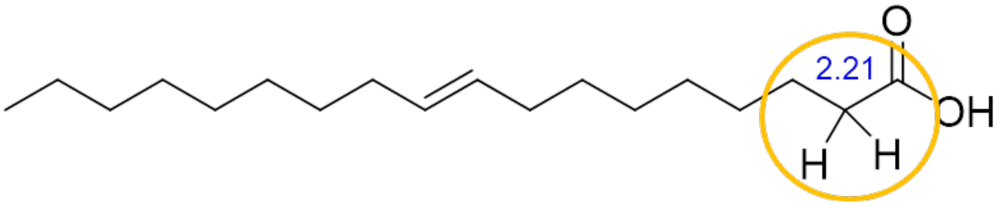

Scheme 1. The chemical structure of oleic acid and the literature chemical shift value from α-protons.[9]

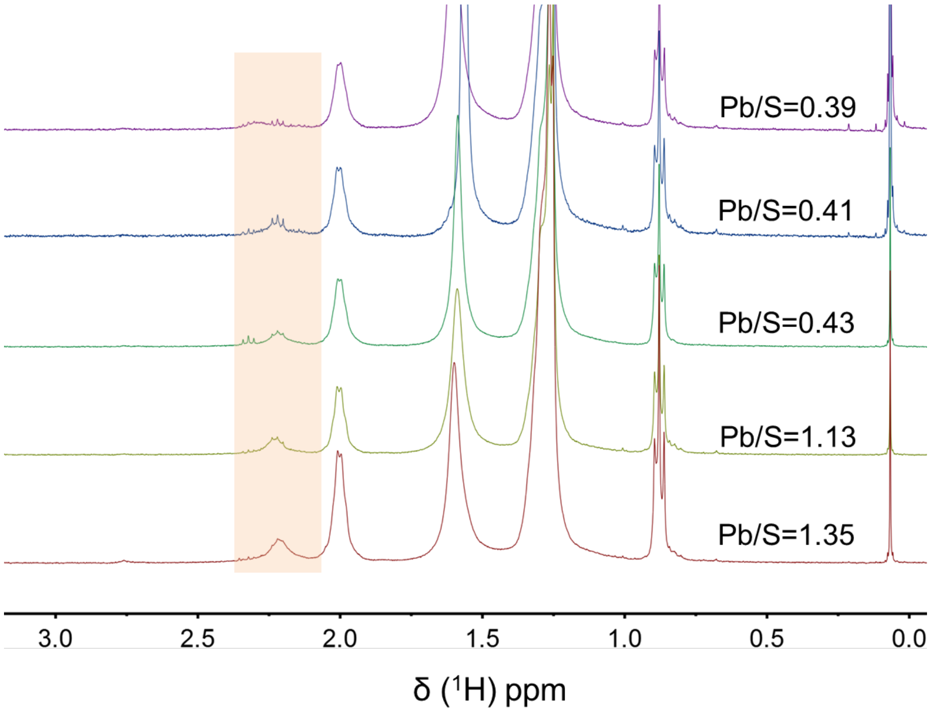

Figure S7. ¹H NMR analysis of PbS NC aliquots dispersed in $CDCl_3$ at different Pb/S ratios. The highlighted area indicates the triplet signals associated with the secondary carbon protons which were adjacent to the carbonyl moiety. The highlighted area shows that the population of protons adjacent to the carbonyl moiety was significantly decreased in the S-rich NCs.

Band Structure Analysis

Ultraviolet photoelectron spectroscopy (UPS) measurements were performed on a KRATOS Inc. AXIS – NOVA spectrometer with a He (I) (21.2 eV) discharge lamp as an incident photon source. All the UPS and XPS samples were prepared by depositing three layers of PbS NCs onto pre-cleaned ITO substrates.

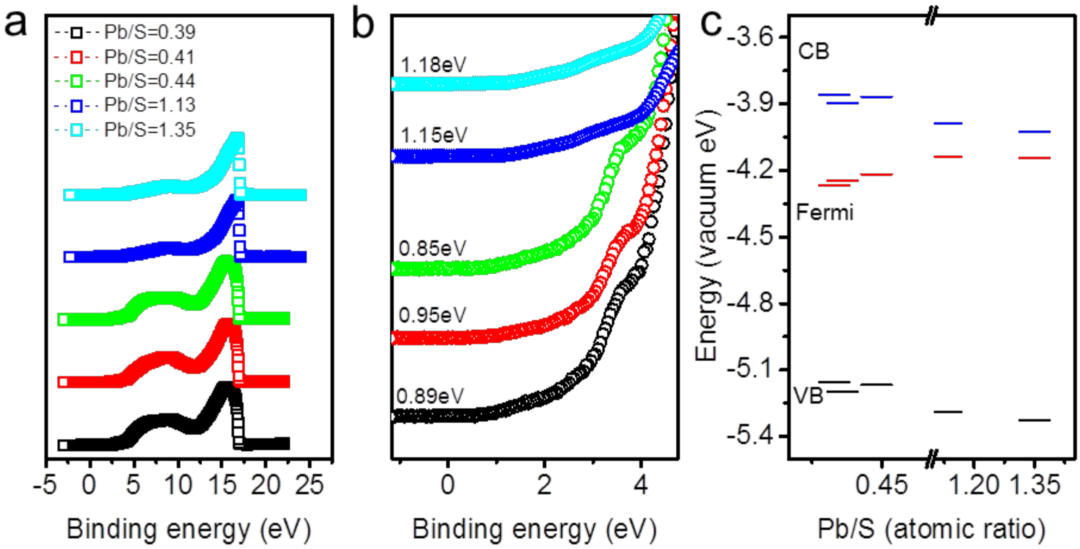

Figure S8. UPS spectra of the 1.3 eV PbS NCs film on the top of ITO slides. a) Full range spectra, b) shows magnified spectra near the Fermi edge. Spectra were shifted for clarity, and the identified binding energy values are highlighted beside each plot. Distribution of c) Valence band edge (VB), Fermi level (Fermi) and Conduction band edge (CB) energy levels as a function of the Pb/S ratio.

Optical Property Analysis

The absorption spectrum was measured by employing a Cary 5000 UV-vis-NIR spectrometer and the band gap value of PbS NCs was estimated from the wavelength at the first exciton peak. Steady-state photoluminescence (PL) measurements were performed on a Fluorolog Horiba Jobin Yvon Spectrofluorometer with a 450 W xenon lamp excitation source and NIR detector. Time-resolved photoluminescence (TRPL) measurements were carried out by employing a FluoTime 300 PicoQuant GmbH time-correlated single photon counting apparatus equipped with an H10330A-45 Hamamatsu NIR-PMT detector. All the PL samples were prepared by depositing six layers of PbS NCs onto pre-cleaned ITO substrates capped by ZnO layers.

SIII Device Characterizations

Solar cell J-V curves were recorded using a Keithley 2400 and a LOT Quantum Design solar simulator (LSE340/1/850.27C). It equipped with 300 W Xe lamp and an AM1.5 filter as a light source. The photon flux was calibrated by a silicon reference cell (RERA SOLUTIONS, RQS4695) before each

measurement. External quantum efficiency (EQE) measurements were performed on a SpeQuest QE system. The system was equipped with a 100W quartz tungsten halogen light together with 150mm F/4.2 monochromator as irradiance source. An SR830 DSP Lock-In Amplifier (locked to 83 Hz light chopper) and a Melles Griot IV converter were employed to record the photocurrents. The effective wavelength range was from 350 nm to 1800 nm and the entire test was calibrated by NIST traceable Silicon (200 nm-1100 nm) and Germanium (700-1800 nm) reference cells with 0.0134 cm^2 area pre-defined masks.

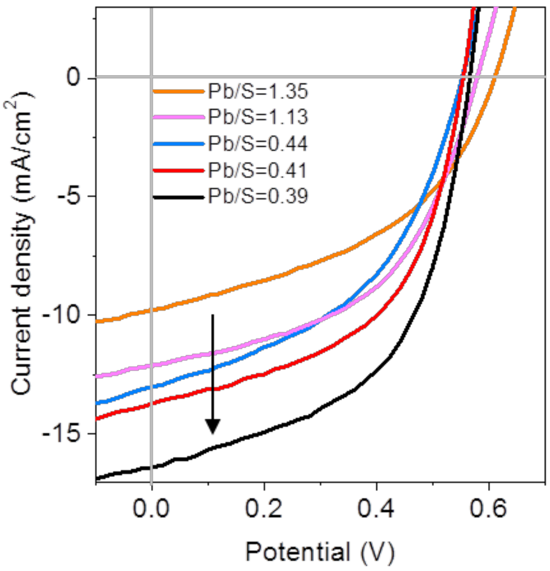

Figure S9. Typical J-V curves of the 1.3 eV PbS NCSCs with a Pb/S ratio range from 1.35 to 0.39, and employing TBAI as the only ligands. The arrow indicates the direction of increasing J_{sc}.

The ideality factor n values were extracted from fitting the simple diode equation (equation 1)[10] and also verified based on solving the diode equation (equation 2) through conductance derivative method.[11]

i. Simple diode equation fitting approach:

The simple diode equation is as below:

$$I = I_0 \left[\exp\left(\frac{qV}{nk_BT}\right) - 1\right] \quad \text{(Equ. 1)}$$

where I_0 is the saturation current density, q is the electronic charge, V is the potential drop across the junction, n is the ideality factor, k_B is Boltzmann's constant, and T is the temperature. The n value was obtained from fitting the diode equation to measured curves at 300 K. When V > 50 - 100 mV the -1 term can be ignored and so the equation can be simplified to:

$$I = \mathrm{I}_0 \left[\exp\left(\frac{\mathrm{qV}}{\mathrm{n}k_B T}\right)\right] \quad \text{(Equ. 2)}$$

Taking the natural logarithm of both sides of the equation gives:

$$\ln I = \ln I_0 + \frac{\mathrm{qV}}{\mathrm{n}k_B T} \quad \text{(Equ. 3)}$$

When plotting the natural logarithm of the current against the voltage, the slope gives $\frac{\mathrm{q}}{\mathrm{n}k_B T}$ and the intercept gives $\ln I_0$. The ratio $\frac{k_B T}{\mathrm{q}}$ is 25.87 mV at 300 K.

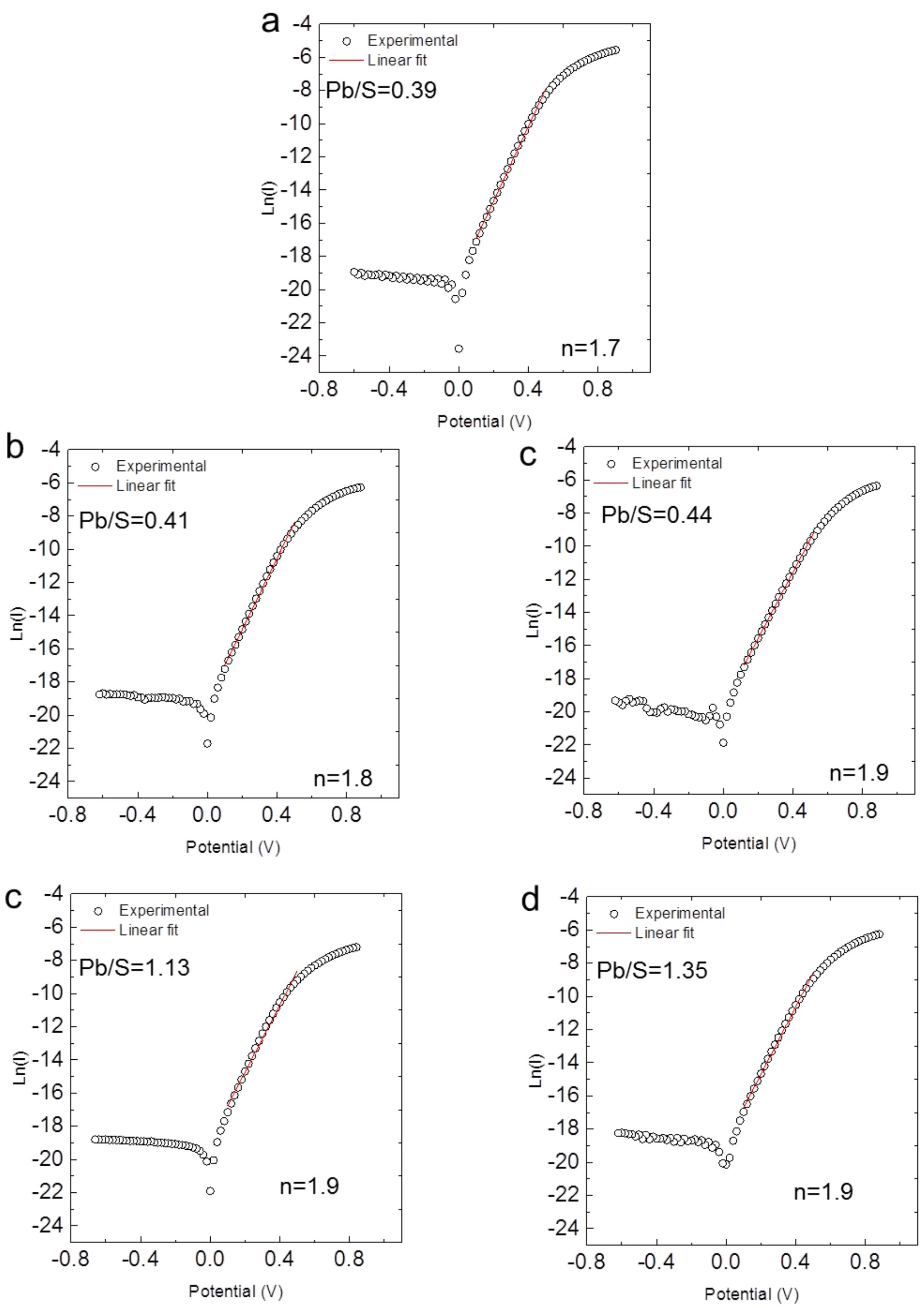

Figure S10. n values estimated from fitting to the exponential portion of the forward bias currents based on the simple diode equation (Equ.3) and the potential range was fixed from 0.1 V to 0.5 V among all the analysis.

ii. Conductance derivate method (Werner approach):

The equations used as follows:

$$I = I_s \left[\exp\left(\frac{q}{nkT}(V - IR_s)\right) - 1\right] + G_{sh}(V - R_s I) \quad \text{(Equ. 4)}$$

The conductance (Equation 5):

$$G = \frac{\partial I}{\partial V} \text{ (Equ. 5)}$$

The shunt conductance G_{sh} was evaluated from the reverse bias curve by simple linear fitting. The values of the G_{sh} gives the shunt current through Equation 6:

$$I_P = G_{sh}\text{V (Equ. 6)}$$

After correcting the current from the shunt current and obtain the current across the diode under V-R_sI>>kT condition, the Equation 4 can be written as:

$$I_{cor} = I_s \left[\exp\left(\frac{\beta}{n}(V - IR_s)\right)\right] \text{ (Equ. 7)}$$

Based on equation 5, the corrected conductance of the diode was obtained as:

$$G_{cor} = \frac{\beta}{n} I_{cor}(1 - G_{cor} R_s) \text{ (Equ. 8)}$$

Equation 8 can be transformed as:

$$\frac{1}{G_{cor}} = \frac{\partial V}{\partial I_{cor}} = \frac{1}{I_{cor}} \frac{\partial V}{\partial \ln I_{cor}} \text{ (Equ. 9)}$$

The equation 9 can be written as:

$$\frac{\partial V}{\partial \ln I_{cor}} = \frac{n}{\beta} + R_s I_{cor} \text{ (Equ. 10)}$$

Based on equation 10, the dark I-V curves can be plotted as $\frac{\partial V}{\partial \ln I_{cor}}$ against I_{cor} and the linear fitting of the plots give the y-axis intercept $\frac{n}{\beta}$ and a slope R_s.

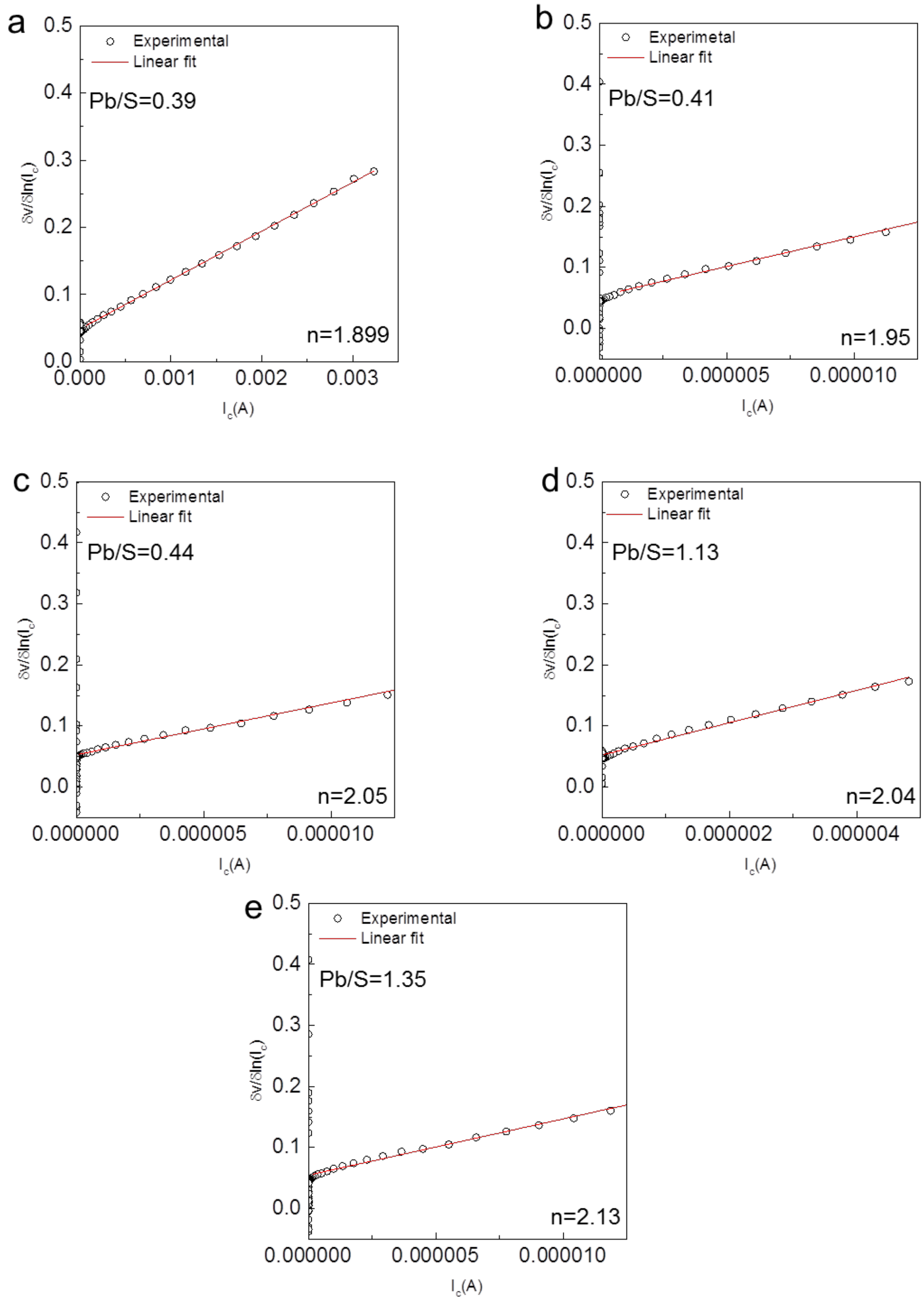

Figure S11. n values estimated from fitting to the linear portion of the forward bias corrected currents based on derivate solved diode equation (Equ.10).

Table S4. Summary of V_{oc}, J_{sc}, R_s, R_{sh}, FF, n and PCE average values of as-prepared solar cells correlated with NCs Pb/S ratio, n values were calibrated based on Equation 3 and Equation 10. Each NCSC result was averaged across 9 samples on 3 different substrates and employing TBAI as the only ligand.

Pb:S	V_{oc}	J_{sc}	R_s	R_{sh}	FF	n	PCE
0.39	0.55±0.01	15.39±0.51	4.47±0.9	130.26±29.26	0.52±0.02	1.823±0.027	4.66±0.32
0.41	0.55±0.01	13.32±0.37	5.62±1.3	126.95±20.51	0.51±0.02	1.910±0.027	3.72±0.19
0.44	0.58±0.01	13.01±0.67	3.65±1.0	87.28±4.94	0.52±0.01	2.000±0.017	3.33±0.13
1.13	0.57±0.01	12.11±0.31	7.24±1.4	88.85±12.79	0.45±0.01	2.003±0.022	3.28±0.09
1.35	0.59±0.01	8.98±0.33	5.97±1.4	63.60±4.80	0.44±0.01	2.04±0.0329	2.42±0.17

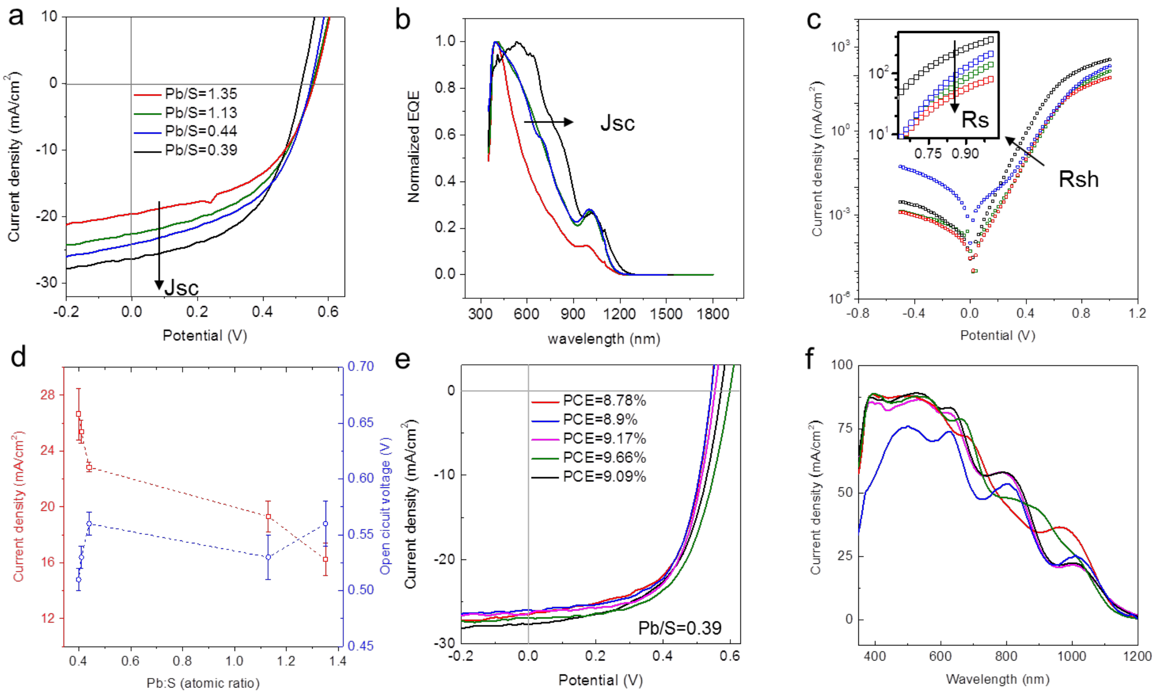

Figure S12. Typical J-V curves a) and normalized EQE spectra of 1.3eV PbS NCSCs with Pb/S ratio range from 1.35 to 0.39 and employing EDT as an electron-blocking layer. Arrows indicate the direction of increasing Jsc. c) Log current density–voltage curves for dark diode behavior for as-prepared 1.3eV PbS NCSCs. Arrows indicate the direction of increasing Rs and Rsh. d) Open circuit voltages (V_{OC}) and short-circuit current densities (J_{sc}) as a function of the Pb/S ratio. e, f) Additional J-V curves and EQE spectra from hydrophilic PbS NCSC (Pb/S = 0.39) with high PCE performance. The current density verification is summarized in Table S5.

Table S5. Summary of the V_{oc}, J_{sc}, R_s, R_{sh}, FF and PCE average values of as-prepared TBAI/EDT structure solar cells correlated with the NCs Pb/S ratio, champion devices are quoted in the brackets. Each NCSC result was averaged across 9 samples on 3 different substrates. Sample *a* is the world-record PbS NCs solar cell with similar device structure but were made from employing Perovskite precursors as surface ligands and polymer PD2FCT-29DPP as the hole transport layer.[12] Sample *b* was the first high-efficiency PbS NC solar cell based on TBAI/EDT structure with PCE above 8%.[13] Sample *c* was another example of employing TBAI/EDT structure for high-efficiency PbS NC solar cell but with external additives on ZnO layers.[14]

**Pb:S ratio**	**V_{oc} (V)**	**J_{sc} (mA/cm^2)**	**R_s (Ωcm^2)**	**R_{sh} (Ωcm^2)**	**FF**	**PCE (%)**
0.39	0.51±0.01	26.65±1.85 (28.5)	7.29±1.66	329.71±22.45	0.57±0.02	8.57±0.55 (9.33)
0.41	0.53±0.01	25.38±0.83 (26.21)	8.84±0.82	254.47±14.90	0.54±0.02	7.14±0.23 (7.43)
0.44	0.56±0.01	22.85±0.35 (23.2)	8.33±0.47	184.56±7.76	0.48±0.01	6.01±0.10 (6.07)
1.13	0.53±0.02	19.32±1.10 (20.42)	10.10±2.51	164.15±18.83	0.46±0.032	4.70±0.54 (5.27)
1.35	0.56±0.02	16.23±3.16 (19.39)	8.08±1.95	127.78±11.68	0.46±0.029	4.16±0.93 (5.19)
a	0.66	30.3	_	_	0.71	14.0%
b	0.5546±0.0055	24.2±0.7	_	_	0638±0.013	8.55±0.18
c	0.67 ± 0.02	21.42 ± 0.72	16.78	601	0.62 ± 0.04	8.82 ± 0.56

Table S6. Comparisons of the current density mismatch between quantum efficiency analysis system and solar simulator system on the as-prepared hydrophilic PbS NCSCs, data were taken from Figure S12 e and f. The current density of EQE system was integrated under AM1.5G TILT (ASTM-G173-03) with solar cell mask area 0.0134 cm^2.

Pb:S ratio	Sample curve color	J_{sc} (mA/cm^2)	J_{sc} (EQE) (mA/cm^2)	Mismatch (mA/cm^2)
0.39	Black	27.67	25.922	1.748
0.39	Green	26.93	25.862	1.068
0.39	Red	26.53	25.702	0.828
0.39	Pink	26.39	25.215	1.175
0.39	Blue	26.00	25.59	0.41

(1) Hou, B.; Cho, Y.; Kim, B. S.; Hong, J.; Park, J. B.; Ahn, S. J.; Sohn, J. I.; Cha, S.; Kim, J. M. Highly Monodispersed PbS Quantum Dots for Outstanding Cascaded-Junction Solar Cells. ACS Energy Letters 2016, 1 (4), 834-839. DOI: 10.1021/acsenergylett.6b00294.

(2) Hou, B.; Kim, B.-S.; Lee, H. K. H.; Cho, Y.; Giraud, P.; Liu, M.; Zhang, J.; Davies, M. L.; Durrant, J. R.; Tsoi, W. C.; et al. Multiphoton Absorption Stimulated Metal Chalcogenide Quantum Dot Solar Cells under Ambient and Concentrated Irradiance. Advanced Functional Materials 2020, 30 (39), 2004563. DOI: https://doi.org/10.1002/adfm.202004563.

(3) Kresse, G.; Furthmüller, J. Efficient iterative schemes for ab initio total-energy calculations using a plane-wave basis set. Physical Review B 1996, 54 (16), 11169-11186. DOI: 10.1103/PhysRevB.54.11169.

(4) Jain, A.; Hautier, G.; Moore, C. J.; Ping Ong, S.; Fischer, C. C.; Mueller, T.; Persson, K. A.; Ceder, G. A high-throughput infrastructure for density functional theory calculations. Computational Materials Science 2011, 50 (8), 2295-2310. DOI: https://doi.org/10.1016/j.commatsci.2011.02.023. Kresse, G.; Joubert, D. From ultrasoft pseudopotentials to the projector augmented-wave method. Physical Review B 1999, 59 (3), 1758-1775. DOI: 10.1103/PhysRevB.59.1758. Blöchl, P. E. Projector augmented-wave method. Physical Review B 1994, 50 (24), 17953-17979. DOI: 10.1103/PhysRevB.50.17953.

(5) Perdew, J. P.; Ruzsinszky, A.; Csonka, G. I.; Vydrov, O. A.; Scuseria, G. E.; Constantin, L. A.; Zhou, X.; Burke, K. Restoring the Density-Gradient Expansion for Exchange in Solids and Surfaces. Physical Review Letters 2008, 100 (13), 136406. DOI: 10.1103/PhysRevLett.100.136406.

(6) Mortensen, J. J.; Hansen, L. B.; Jacobsen, K. W. Real-space grid implementation of the projector augmented wave method. Physical Review B 2005, 71 (3), 035109. DOI: 10.1103/PhysRevB.71.035109. Enkovaara, J.; Rostgaard, C.; Mortensen, J. J.; Chen, J.; Dułak, M.; Ferrighi, L.; Gavnholt, J.; Glinsvad, C.; Haikola, V.; Hansen, H. A.; et al. Electronic structure calculations with GPAW: a real-space implementation of the projector augmented-wave method. Journal of Physics: Condensed Matter 2010, 22 (25), 253202. DOI: 10.1088/0953-8984/22/25/253202.

(7) Larsen, A. H.; Vanin, M.; Mortensen, J. J.; Thygesen, K. S.; Jacobsen, K. W. Localized atomic basis set in the projector augmented wave method. Physical Review B 2009, 80 (19), 195112. DOI: 10.1103/PhysRevB.80.195112.

(8) Kuisma, M.; Ojanen, J.; Enkovaara, J.; Rantala, T. T. Kohn-Sham potential with discontinuity for band gap materials. Physical Review B 2010, 82 (11), 115106. DOI: 10.1103/PhysRevB.82.115106. Castelli, I. E.; Olsen, T.; Datta, S.; Landis, D. D.; Dahl, S.; Thygesen, K. S.; Jacobsen, K. W. Computational screening of perovskite metal oxides for optimal solar light capture. Energy & Environmental Science 2012, 5 (2), 5814-5819, 10.1039/C1EE02717D. DOI: 10.1039/C1EE02717D.

(9) Knothe, G.; Kenar, J. A. Determination of the fatty acid profile by 1H-NMR spectroscopy. Eur. J. Lipid Sci. Technol. 2004, 106 (2), 88-96. DOI: 10.1002/ejlt.200300880.

(10) Clifford, J. P.; Johnston, K. W.; Levina, L.; Sargent, E. H. Schottky barriers to colloidal quantum dot films. Appl. Phys. Lett. 2007, 91 (25), 253117. DOI: doi:http://dx.doi.org/10.1063/1.2823582. Sze, S. M. N., KWOK K. Physics of Semiconductor Devices; John Wiley & Sons, 2007.

(11) Werner, J. H. Schottky barrier and pn-junctionI/V plots — Small signal evaluation. Applied Physics A 1988, 47 (3), 291-300. DOI: 10.1007/BF00615935.

(12) Kim, H. I., Baek, S.-W., Cheon, H. J., Ryu, S. U., Lee, S., Choi, M.-J., Choi, K., Biondi, M., Hoogland, S., de Arquer, F. P. García, Kwon, S.-K., Kim, Y.-H., Park, T., Sargent, E. H., A Tuned Alternating D–A Copolymer Hole-Transport Layer Enables Colloidal Quantum Dot Solar Cells with

Superior Fill Factor and Efficiency. Adv. Mater. 2020, 32, 2004985. https://doi.org/10.1002/adma.202004985

(13) Chuang, C.-H. M.; Brown, P. R.; Bulović, V.; Bawendi, M. G. Improved performance and stability in quantum dot solar cells through band alignment engineering. Nat Mater 2014, 13 (8), 796-801, Letter. DOI: 10.1038/nmat3984 http://www.nature.com/nmat/journal/v13/n8/abs/nmat3984.html#supplementary-information.

(14) Azmi, R.; Oh, S.-H.; Jang, S.-Y. High-Efficiency Colloidal Quantum Dot Photovoltaic Devices Using Chemically Modified Heterojunctions. ACS Energy Lett. 2016, 1 (1), 100-106. DOI: 10.1021/acsenergylett.6b00070.

(15) Madsen, J.; Susi, T. The AbTEM Code: Transmission Electron Microscopy from First Principles. *Open Res Europe* **2021**, *1*, 24. https://doi.org/10.12688/openreseurope.13015.1.

(16) Lobato, I.; Dyck, D. V. An Accurate Parameterization for the Scattering Factors, Electron Densities and Electrostatic Potentials for Neutral Atoms That Obey All Physical Constraints. *Acta Crystallographica Section A* **2014**, *70*, 636–649.

(17) Ophus, C. A Fast Image Simulation Algorithm for Scanning Transmission Electron Microscopy. *Advanced Structural and Chemical Imaging* **2017**, *3*.
